# The potential role of the antioxidant and detoxification properties of glutathione in autism spectrum disorders: a systematic review and meta-analysis

**DOI:** 10.1186/1743-7075-9-35

**Published:** 2012-04-24

**Authors:** Penelope AE Main, Manya T Angley, Catherine E O'Doherty, Philip Thomas, Michael Fenech

**Affiliations:** 1 Sansom Institute for Health Research, University of South Australia, City East Campus, Adelaide, SA 5000, Australia; 2 Food and Nutritional Sciences, Commonwealth Scientific and Industrial Research Organisation, Kintore Ave, Adelaide, SA 5000, Australia

**Keywords:** γ-glutamyl cycle, Trans-sulphuration pathway, Metabolites, Genes, Supplementation, Autism spectrum disorders

## Abstract

**Background:**

Glutathione has a wide range of functions; it is an endogenous anti-oxidant and plays a key role in the maintenance of intracellular redox balance and detoxification of xenobiotics. Several studies have indicated that children with autism spectrum disorders may have altered glutathione metabolism which could play a key role in the condition.

**Methods:**

A systematic literature review and meta-analysis was conducted of studies examining metabolites, interventions and/or genes of the glutathione metabolism pathways i.e. the γ-glutamyl cycle and trans-sulphuration pathway in autism spectrum disorders.

**Results:**

Thirty nine studies were included in the review comprising an *in vitro *study, thirty two metabolite and/or co-factor studies, six intervention studies and six studies with genetic data as well as eight studies examining enzyme activity.

**Conclusions:**

The review found evidence for the involvement of the γ-glutamyl cycle and trans-sulphuration pathway in autistic disorder is sufficiently consistent, particularly with respect to the glutathione redox ratio, to warrant further investigation to determine the significance in relation to clinical outcomes. Large, well designed intervention studies that link metabolites, cofactors and genes of the γ-glutamyl cycle and trans-sulphuration pathway with objective behavioural outcomes in children with autism spectrum disorders are required. Future risk factor analysis should include consideration of multiple nutritional status and metabolite biomarkers of pathways linked with the γ-glutamyl cycle and the interaction of genotype in relation to these factors.

## Background

Autism spectrum disorders are a heterogeneous group of neurodevelopmental conditions comprising autistic disorder which is characterised by impairments in reciprocal social interaction and communication and the presence of stereotyped behaviours, Asperger's Syndrome which is distinguished by no significant delay in early language acquisition or cognitive abilities, and pervasive developmental disorder - not otherwise stated (PDD-NOS) in which individuals do not fully meet the criteria for autistic disorder or Asperger's syndrome. Over the last 30 years the number of diagnosed cases has increased from 0.4-0.5 to 4.0 per 1000 for autistic disorder and from 2 to 7.7-9.9 per 1000 for autism spectrum disorders [1-3] which is largely attributable to broadening diagnostic criteria, younger age at diagnosis and improved case ascertainment [4]. Autism spectrum disorders are increasingly being recognised as a major public health issue.

While the exact cause of autism is unknown, a strong genetic component has been identified as shown by family and twin studies which have found concordance rates of 82-92% in monozygotic twins compared with 1-10% in dizygotic twins, sibling recurrence risk at 6-8% and heritability estimates of > 90% [5,6]. Recent studies have shown that autistic disorder is likely to involve multiple genes [7-9] although a common genetic change is not seen in all cases suggesting that it is likely to be a cluster of conditions, each with its own individual and yet overlapping pathology. Environmental factors such as heavy metal toxicity [10-12], sub-clinical viral infections [13] and gastro-intestinal pathology [14,15], as well as endogenous toxins produced by metabolic processes [16], hormones (reviewed in [17]) and gastro-intestinal bacteria [18,19] have also been suggested as playing a role in the aetiology of the disorder, although none of these have been thoroughly investigated. Large, well designed studies, such as the Childhood Autism Risks from Genetics and Environment (CHARGE) [20], are currently underway to further elucidate the role of genes and environment.

Cellular detoxification systems are of critical importance in providing protection against the effects of endogenous and exogenous toxins. Glutathione redox and the glutathione-s-transferases reviewed below constitute one such system.

### Glutathione redox and autism spectrum disorders

Glutathione (L-γ-glutamyl-L-cysteinyl-glycine) is an intracellular peptide that has a wide range of functions including detoxification of xenobiotics and/or their metabolites [21,22], maintenance of the intracellular redox balance [23], and is the major endogenous antioxidant produced to combat free radical insults [24-26]. Other metabolic functions include cysteine storage [21], signal transduction [27] and apoptosis [28].

**Figure 1:**
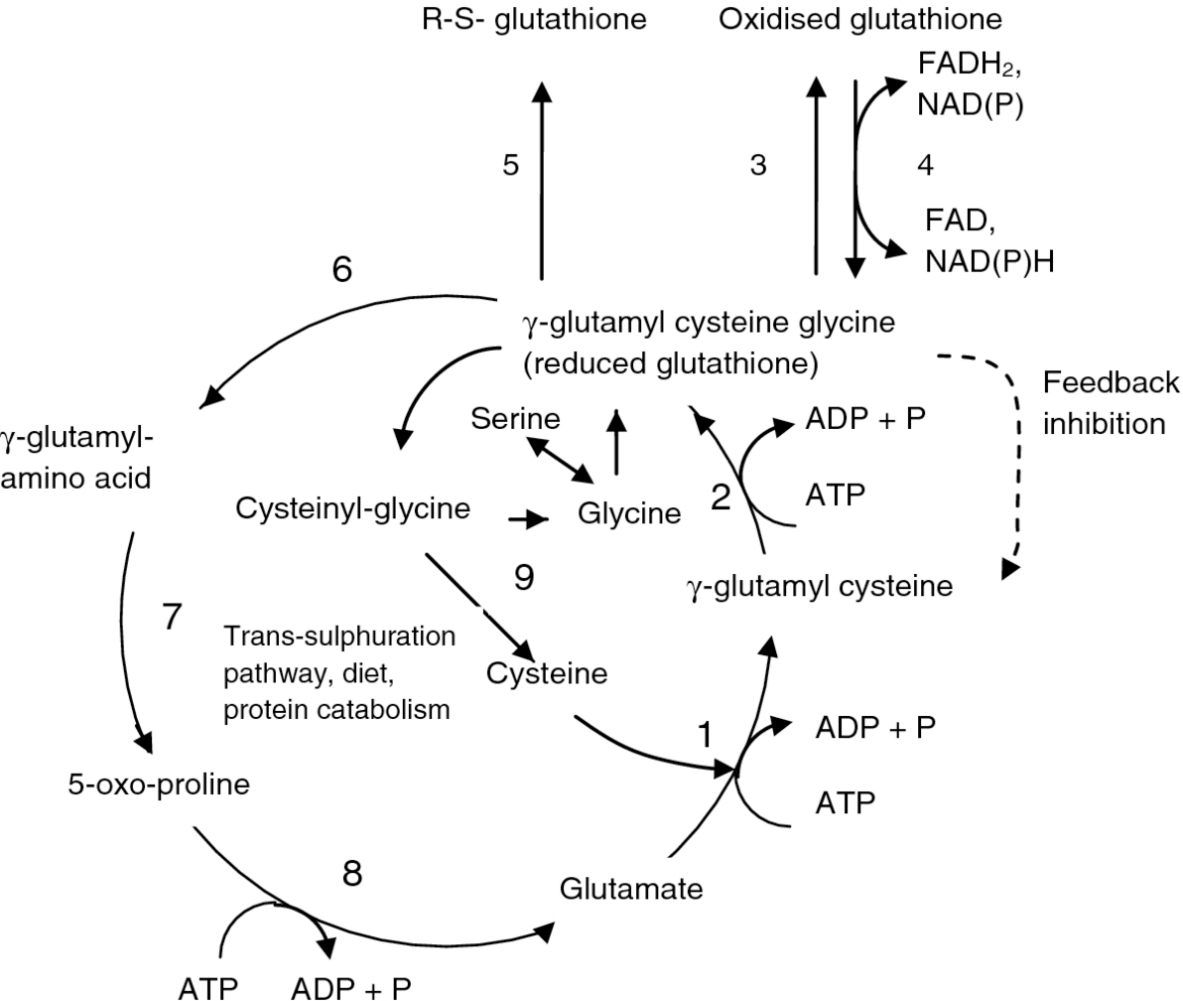
**γ-Glutamyl cycle, 1 Glutamate cysteine ligase (GCL), 2 Glutathione synthetase (GS), 3 Glutathione peroxidase, 4 Glutathione reductase, 5 Glutathione-S-transferases (detoxification reactions), 6 γ-glutamyl-transferase (GGT), 7 γ-glutamyl cyclotransferase (GCT), 8 5-Oxoprolinase, 9 Dipeptidase, R Protein, FAD, FADH_2 _Flavin-adenine dinucleotide, NAD, NAD^+ ^Nicotinamide-adenoside dinucleotide, NADP, NADPH^+ ^Nicotinamide-adenoside dinucleotide phosphate, ADP, ATP Adenosine diphosphate, Adenosine triphosphate**.

Within the cell, approximately 90% of glutathione is located in the cytosol, 10% in the mitochondria and a small percentage in the endoplasmic reticulum [29]. Approximately 85% of total cellular glutathione is free and unbound whilst the rest is bound to proteins [30]. Glutathione is synthesised in the cytosol in two steps (Figure 1).

The first step of glutathione synthesis involves the formation of glutamylcysteine from glutamate and cysteine in an ATP dependent reaction catalysed by glutamate-cysteine-ligase (GCL) which requires either Mg ^2+ ^or Mn ^2+ ^as a cofactor. This is considered to be the rate limiting step because it is dependent on the bioavailability of cysteine and the activity of GCL, the latter of which is modified by competitive inhibition by reduced glutathione (GSH) [31-34]. In the second step, glutathione synthetase (GS) adds glycine to glutamyl-cysteine to form glutathione (γ-glutamyl-cysteinyl-glycine).

More than 98% of total glutathione is present as GSH and the rest is found as the oxidised form, glutathione disulfide (GSSG) or a range of glutathione-S-conjugates. GSH is readily converted to GSSG by the seleno-enzyme glutathione peroxidase (GPx) during periods of oxidative stress, and is reverted to the reduced form by glutathione reductase (GSH-R) [35]. GSH is also important in detoxification as it is used to conjugate a wide variety of exogenous compounds including carcinogens, toxins and drugs and endogenous electrophiles. The glutathione conjugate is subsequently secreted from the cell [36].

Glutathione degradation takes place in the extracellular space. Cysteine is released from extracellular glutathione by γ-glutamyl-transferase (GGT) located on the apical surface of the kidney, intestine and the epithelia of most transporting ducts, including the liver and bile ducts [37]. Expression of GGT is tissue and developmental stage specific and its activity may be induced by certain xenobiotics [37]. GGT hydrolyses the γ-glutamyl bond of glutathione or glutathione-S-conjugates and transfers the γ-glutamyl moiety to an acceptor molecule, often an amino acid [38]. If the substrate is glutathione, cysteinyl-glycine is released and subsequently cleaved into cysteine and glycine by cell surface dipeptidases. The γ-glutamyl amino acid can be transported back into the cell where γ-glutamyl cyclo-transferase (GCT) releases the acceptor amino acid to form 5-oxo-proline, the latter of which is converted back to glutamate by oxo-prolinase and used for GSH synthesis.

**Figure 2:**
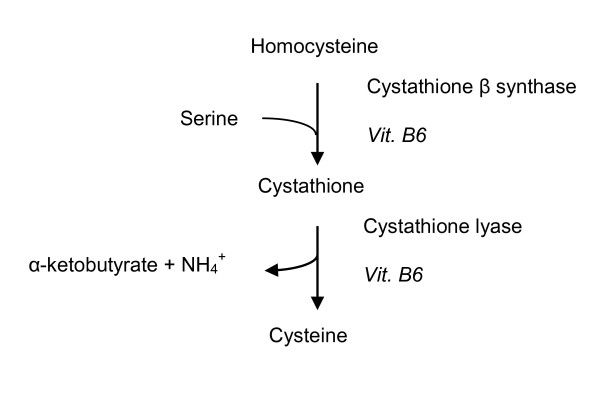
**Trans-sulphuration pathway Vit**. B6 Vitamin B6.

About half the cysteine used for glutathione synthesis is produced by the trans-sulphuration pathway [33]. The trans-sulphuration pathway involves conversion of homocysteine to cystathione and ultimately to cysteine in two vitamin B6 dependent reactions catalysed by cystathione-β-synthase and cystathione lyase respectively (Figure 2). The remainder is obtained through the diet and protein catabolism. The trans-sulphuration pathway is closely linked to the folate-methionine cycle and is particularly active in the liver and absent or less active in other tissues, the foetus, neonates and in patients with homocysteinemia [39]. Neurones depend on glial cysteine for glutathione synthesis as they lack the trans-sulphuration pathway which in turn results in them being more susceptible to oxidative stress [40].

Glutathione status is an accurate indicator of cell functionality and viability [41-43]. The ratio of GSH:GSSG (glutathione redox ratio) is a sensitive index of oxidative stress, which can lead to a toxic imbalance between the production and removal of reactive oxygen species (ROS). A shift in the glutathione redox ratio towards the oxidised state may lead to decreased cell proliferation, DNA damage [44] and increased apoptosis [45] that could potentially affect neurological development in the early stages of life. As a decreased glutathione redox ratio has also been reported in many studies of individuals with autistic disorder [46-50], it may be hypothesised that a shift in the glutathione redox ratio may play a role in the aetiology of autism. This article systematically reviews the evidence for a role of glutathione redox in the aetiology of autism spectrum disorders and considers the research questions:

1) Is there an association between metabolites/co-factors/genes/enzymes of the γ-glutamyl cycle or trans-sulphuration pathway and autism spectrum disorders?

2) If so, does normalisation of metabolite levels of the γ-glutamyl cycle or trans-sulphuration pathway lead to clinically significant improved outcomes for children with autism spectrum disorders?

## Methods

### Selection of studies for review

The inclusion criteria for studies were defined as:

(a) participants diagnosed with an autism spectrum disorder using standardised criteria such as the American Psychiatric Association's Diagnostic and Statistical Manual of Mental Disorders (DSM)-IV-R [51] or the Childhood Autism Rating Scale (CARS) scores [52]; and

(b) data for metabolites, co-factors, genes and/or enzymes associated with the γ-glutamyl cycle or the trans-sulphuration pathway, and/or

(c) interventions using metabolites or cofactors of the γ-glutamyl cycle or the trans-sulphuration pathway.

(d) full text English language articles published between 1970 and November 2011.

### Information retrieval

Information retrieval was performed using the following electronic databases: Embase, Medline, Cinahl, Scopus, Web of Science and International Pharmaceutical Abstracts (search terms in the Supplementary On-line Material). In addition, studies were identified from the reference lists of obtained published articles, editorials and known studies. Authors were contacted if not enough data was included in the original manuscript for analysis, for clarification of terms or to confirm whether the article contained data previously published by the same research group.

All potential studies identified were independently evaluated for inclusion by two primary reviewers. The primary reviewers were not blinded to the authors, institutions or source of publication at any time during the selection process. Disagreements about the inclusion/exclusion of studies were discussed and consensus achieved. Provision was made for a third reviewer if consensus was unattainable but did not prove necessary. When multiple papers from a single study had been published, we used the latest publication and supplemented it with data from the earlier publication(s).

### Data extraction and methodological quality assessment

Data extraction for each included study was performed by PM and checked by two primary reviewers (MA, CO'D). Differences were resolved by consensus. The Newcastle Ottawa Scale [53] for case control studies was modified to assess the methodological quality of observational articles for the review (Table 1). The quality of each article was independently assessed by two primary reviewers (PM and COD) and assigned a score. Using a similar process, the risk of bias for intervention trials included in the review omitting the case report [54] was assessed using the criteria set out in the Cochrane Collaboration Handbook [55]. In addition, a level of evidence was assigned to each study using the Australian National Health and Medical Research Council criteria (Table 2) [56].

**Table 1:** Modified Newcastle Ottawa Scale

1. Selection		
Case definition	Yes, with independent validation	2

	Yes, record linkage/self report	1

	No description	0

Representativeness	Consecutive cases	1

	Potential for selection bias/not stated	0

Selection of controls	Community	2

	Hospital/clinic/school	1

	Potential for selection bias/not stated	0

Definition of controls	No family history of autism spectrum disorder	2

	Healthy/other psych/developmental/genetic	1

	disorder	

	Poorly defined/not stated	0

**2. Comparability of cases and controls on the basis of the design**		

Study controls for age		1

Study controls for gender		1

**3. Exposure**		

Ascertainment of exposure		

	Laboratory blinded to case/control status	1

	Laboratory unblinded/not stated	0

Method of ascertainment same for cases and controls		

	Yes	1

	No	2

**4. Additional criteria for genetic studies**		

a. Consideration of Hardy Weinberg Equilibrium		

	Yes	1

	No	0

b. Power calculations		

	Yes	1

	No	0

c. Correction for multiple comparisons		

	Yes	1

	No	0

d. Adjustment for population stratification		

	Yes	1

	No	0

**Table 2:** Australian National Health and Medical Research Council Designated Levels of Evidence^1^

Level of evidence	Description
**I**	Evidence obtained from a systematic review of all relevant randomised controlled trials.

**II**	Evidence obtained from at least one properly designed randomised controlled trial.

**III - 1**	Evidence obtained from well designed pseudo-randomised controlled trials (alternate allocation or some other method).

**III - 2**	Evidence obtained from comparative studies with concurrent controls and allocation not randomised (cohort studies), case control studies or interrupted time series with control group.

**III - 3**	Evidence obtained from comparative studies with historical control, two or more single arm studies, or interrupted time series without a parallel control group.

**IV**	Evidence obtained from a case series, either post-test or pre-test and post-test.

^1^Data from reference [53]

### Statistical analyses

The kappa coefficient was calculated to assess the level of agreement for the quality scores between the two coders [57]. Statistical heterogeneity was assessed for key metabolites of the γ-glutamyl cycle, trans-sulphuration pathway and GSH:GSSG using the Review Manager 5 (RevMan) statistical software [58]. Duplicated data presented in more than one publication by the same authors was not included in the statistical analysis. Meta-analysis was conducted using a random effects model where heterogeneity was low to moderate (I^2 ^= 0-60%). Where possible, the studies were stratified according to autism spectrum disorder. Standard deviation was calculated using StatSak statistical software prior to analysis using the RevMan program for studies that published the standard error of the mean rather than the standard deviation [59,60]. Studies are reported using the MOOSE (Meta-analyses of observational studies) Statement [61] and the STREGA (STrengthening the REesporting of Genetic Association studies) checklists [62].

## Results

**Figure 3:**
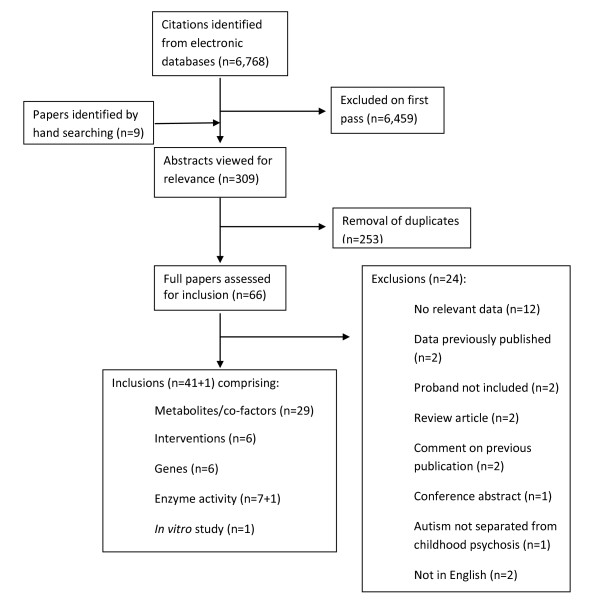
**Flow diagram of research papers retrieved for potential inclusion in our study**.

Sixty six abstracts were identified via the electronic and hand search strategy. Of these, 24 were ineligible for inclusion. Reasons for exclusion were: 1) the paper did not contain any relevant data; 2) the data was already published in another article identified in the search; 3) data did not include the proband with an autism spectrum disorder; 4) the paper was a review article, conference abstract or comment on a previously published article; 5) the authors did not separate data for autism spectrum disorders from other psychological conditions; or 6) they were not English language articles with the exception of a seminal French study widely referred to in English language papers [63] (Figure 3).

Forty two studies were included in the review (41 that met the inclusion criteria plus the French study). Of these, one provided data obtained from *in vitro *models of γ-glutamyl cycle metabolites, twenty nine provided data on metabolites and/or co-factors of the γ-glutamyl cycle or trans-sulphuration pathway, six provided the results of intervention studies, six included genetic data and eight studies provided data on enzyme activity.

An overview of the studies included in this review is presented in Table 3. The level of evidence, study size and ascertainment of cases and controls are indicated along with a quality assessment score and/or assessment of risk of bias. Most studies were of the case control design, however, additionally there were two double blinded [64,65] and one open labelled randomised controlled trial [49], a case series [66] and a case report [54].

**Table 3:** Overview of studies included in the systematic review

Authors (country)	Study size	**Participant characteristics**^**1**^	Ascertainment		Case definition	Outcome measures of interest	Quality Score
			Cases	Controls			
***Level II (Double blinded randomised controlled trial)***							
Bertoglio *et al*. 2010 [64] (USA)	30 cases	Cases 3-8 y, 93%M	Clinical referrals and internal database.	Cross-over study design.	DSM-IV-TR and ADOS plus non-verbal IQ ≥ 49 measured by Wechsler Preschool and Primary Scale of Intelligence, Mullen Scales of Early Learning or Wechsler Intelligence Scale for Children.	Plasma GSH and GSH: GSSG linked to Global Clinical Impressions Score and other objective behavioural measures.	I5
Adams *et al* 2009 [65,67] (USA)	Baseline 77 cases RCT 41 cases (26 intervention, 15 placebo)	Baseline Cases 6.3 (3-8)y, 89.6% M (95% autistic disorder, 3% PDD- NOS, 3% Asperger's Syndrome. RCT Intervention 6.7 y, 92.3%M (96% autistic disorder, 4% Asperger's) Placebo 6.5 y, 93.3%M (100% autistic disorder)	Arizona residents.	Immunosciences reference range for adults.	Previous diagnosis of ASD. No standardised definition.	Erythrocyte glutathione. Behavioural measures.	3* I5
***Open-labelled non-randomised control trial***							
Rossignol *et* *al*. 2007 [49] (USA)	18 cases	Cases 3-16 y, 78%M No seizures. Many supplemented with folinic acid and/or methyl-cobalamin.	Not stated.	Glutathione values from controls in James *et al. *2006	DSM-IV for autistic disorder, CARS	Plasma GSH measured before and after 40 treatments with hyperbaric oxygen. Behavioural measures.	2* I5
***Level III-2 (Casecontrol)***							

Golse *et al*. 1978 [63] (France)	36 cases, 21 controls	Cases 4-19 y, 58%M Controls 5-18 y, 43%M	Referred from 4 clinics in France & 1 from Belgium.	Not stated.	Social isolation, no language, stereotypical behaviour.	Erythrocyte and platelet GPx activity.	2
Rolf *et al*. 1993 [68] (Germany)	18 cases, 14 controls	Cases 9.9 ± 2.8 y (5-14 y) 89%M, medication free. Controls 11.5 ± 2.0 y (8-14 y) 57%M.	Not stated.	Not stated.	DSM-III for autistic disorder.	Platelet glutamic acid.	4
Visconti *et al*. 1994 [69] (Italy)	37 cases (18 with EEG abnormalities), 19 controls	Cases 7 y (3-12 y), 89%M Controls 7 y (3-13 y), 68%M 1 case and 1 control on thioridazine. Fasted.	Patients attending the Department of Child Neurology and Psychiatry, University of Bologna, Italy.	Not stated.	DSM-III-R for autistic disorder.	Serum serine, glutamic acid, glycine and cysteine.	4
D'Eufemia *et* *al*. 1995 [70] (Italy)	40 cases, 46 controls	Cases 12 y 4 mo (7-17 y), 68%M, 32 F Controls 11 y, 2 mo (5-15 y). Medication free for previous month. Fasted.	Referred by the Italian Association of Parents of Autistic Children.	Not stated.	DSM-III-R for autistic disorder.	Serum glutamic acid, glycine and cystine.	5
Yorbik *et al*. 2002 [71] (Turkey)	45 cases, 41 controls	Cases 6.4 ± 2.2 y (4-12 y) 87%M Controls 6.7 ± 2.5 y (4-12 y) 85%M Medication free for previous month and fasted.	Child and adolescent departments of 3 educational hospitals in Ankara.	Children attending the Department of Pediatrics at Gulhane Military Medical School for routine vaccinat- ions.	DSM-IV for autistic disorder.	Plasma and erythrocyte GPx activity.	4
Söğüt *et al*. 2003 [72] (Turkey)	27 cases, 30 controls	Cases 4.7 ± 2.7 y (2-12 y), 59%M Controls 5.1 ± 2.9 y (2-13 y) 53%M Medication free and fasted.	Patients of the Child & Adolescent Psychiatry Dept at Gaziantep University Medical School.	Students at a Gaziantep kindergarten & primary school.	DSM-IV for autistic disorder and CARS > 30.	Plasma GPx activity	5
James *et al*. 2004 [46] (USA)	20 cases (19 regressive autism), 33 controls for the cross sectional study then 8 cases for the intervention.	Cases 6.4 ± 1.5 y, 70%M, 16 supplemented with 400 μg folic acid and 3 μg vitamin B12. Controls 7.4 ± 1.3 y, gender not stated. Supplemented with over the counter multivitamins. Both groups medication free and fasted.	Participants referred to the Arkansas Children's Hospital Research Institute and Dept Pediatrics, University of Arkansas.	Siblings of children with Down syndrome.	DSM-IV for autistic disorder plus diagnostic interview.	Plasma homocysteine, cystathionine, cysteine, tGSH & GSSG measured in all participants. The same parameters were measured before and after 3 mo. folinic acid (800 μg) & betaine (1000 mg) and additional month on same regimen plus vitamin B_12 _(75 μg\kg) for the intervention study.	3 I4
Adams *et al*. 2004 [73] (USA)	24 cases, 11 controls	Cases 4.9 ± 1.4 y, 92%M Controls 6-9 y	Mail out to Greater Phoenix Chapter of the Autism Society of America and the Southwest Autism Research Centre.	Not stated. Appears to be from the same mail out.	Diagnosis of an autism spectrum disorder by a psychiatrist or developmental paediatrician.	Plasma vitamin B_6_.	4
James *et al*. 2006 [47] (USA)	80 cases 73 controls	Cases 7.3 ± 3.2 y (3-14 y), 89% M Controls 10.8 ± 4.1 y, gender not stated. Medication and supplement free. Fasted.	Participants referred from autism clinics of participating physicians in New York and Florida.	Participants of studies of children with Down syndrome or cystic fibrosis. These studies had 53 controls combined including 35 siblings of children with Down's syndrome.	DSM-IV, ADOS or CARS for autistic disorder.	Plasma homocysteine, cystathionine, cysteine, cysteinylglycine, tGSH, fGSH, GSSG.	2
Rose *et al*. 2008 [50] (USA)	242 cases, 75 controls	Cases aged 6.9 ± 2.9 y (3-14 y) Controls aged 10.8 ± 4.1. Gender not stated. Fasted.	Participants referred by the Dennis Develop- mental Center at the University of Arkansas for Medical Sciences and from clinics of participating physicians in New York and Florida.	As for James 2006.	DSM-IV and ADOS or CARS.	tGSH, fGSH, GSSG. Cases stratified by ALAD polymorphism.	2
Pasca *et al*. 2006 [74] (Romania)	12 cases, 9 controls	Cases 8.3 ± 2.8 y, 75% M Controls aged 8.3 ± 1.8 y 66% M Medication and supplement free.	Not stated.	Not stated.	DSM-IV for autistic disorder.	Plasma homocysteine and GPx activity.	3
Shinohe *et al*. 2006 [75] (Japan)	18 cases, 19 controls	Cases 21.2 ± 2.1 y, (18-26 y) 100% M Controls 22.2 ± 2.2 y, (18-26 y) 100%M Medication free.	Advocacy groups in Nagoya and Hamamatsu cities	Recruited through advertisements in Hamamatsu city.	DSM-IV, ADI-R for autistic disorder.	Serum glutamate, glycine and serine. Objective behavioural scores.	9
Adams *et al*. 2006 [76] (USA)	11 cases, 11 controls	Cases 7.2 ± 1.4 y, 73%M Controls 7.8 ± 1.2 y, 91%M Both groups were not supplemented with vitamin B_6 _for the last two months.	Arizona residents	Arizona residents.	Diagnosis of an autism spectrum disorder by a psychiatrist or developmental paediatrician.	Plasma vitamin B_6_.	3
Adams *et al*. 2007 [59] (Australia)	17 cases, 16 controls	Cases 2-16 years. Controls age not stated	Not stated.	Not stated.	DSM-IV for autistic disorder.	Plasma homocysteine.	4
Suh *et al*. 2008 [77] (USA)	31 cases, 11 controls	Cases 4.17 ± 1.3 y, 84%M Controls 6.9 ± 1.6 y, 82%M Medication and supplementation free. Not stated whether fasting.	Pfeiffer Clinic	Pfeiffer Clinic.	DSM-IV, ADI-R for autism spectrum disorders.	Plasma homocysteine, cystathione, cysteine, tGSH, cysteinyl-glycine.	5
Jory & McGinnis 2008 [78] (Canada)	20 cases, 15 controls	Cases 3.9 ± 1.7 y, 80% M Controls 3.87 ± 1.1 y 40% M Medication and supplement free. Fasted.	Not stated. Parent assertion, hard copy confirmation not sought.	Not stated.	DSM-IV for autistic disorder.	Erythrocyte selenium levels.	2
Vojdani *et al*. 2008 [79] (USA)	1027 cases, 113 controls	Cases 2-15 y, 75%M Controls aged 5-15, gender not stated	Participating clinicians from 10 clinics.	Children without autism attending the laboratory for allergy testing who obtained normal results.	DSM-IV and/or ICD-10 For autistic disorder.	Correlation between glutathione and natural killer cell (NK) activity. NK activity and treatment with glutathione.	3
James *et al*. 2009 [80] (USA)	10 case lymphoblastoid cell lines 10 control lymphoblastoid cell lines	Cases 7.8 ± 3.1 y 100%M Controls 27.7 ± 9.1 y 100%M.	AGRE	Coriell Cell Repository. No documented behavioural or neurological disorders.	DSM-IV for autistic disorder	Intracellular glutathione redox status, effect of thimerosal induced and nitrosative oxidative stress on GSH:GSSG.	5
James *et al*. 2009 [48] (USA)	48 cases, 42 controls	Cases 4.8 ± 0.8 y, 82%M Controls 4.5 ± 0.9 y, gender not stated. Supplement free and fasted.	Not stated.	Not stated. No history of developmental delay or neurological symptoms.	DSM-IV for autistic disorder and CARS > 30	Plasma homocysteine, cysteine, cysteinylglycine, tGSH, fGSH, GSSG before and after 3 mo. intervention with folinic acid (400 μg) and vitamin B_12 _(75 μg/kg).	4 I4
Al-Gadani *et* *al*. 2009 [81] (Saudi Arabia)	30 cases, 30 controls	Cases 3-15 y, 73%M Controls 3-15 y, 67%M Medication and supplementation free. Fasted.	Not stated.	Not stated.	DSM-IV for autistic disorder.	Plasma GSH and GSH-Px activity.	4
Pasca *et al*. 2009 [60] (Romania)	15 cases, 13 controls.	Cases 5.1 ± 0.45 y, 87%M Age and gender matched. Supplement free. Fasted.	Not stated.	Not stated.	DSM-IV-R for autistic disorder, PDD-NOS or Asperger's Syndrome.	Plasma homocysteine, cysteine, cystathionine, serine, glycine. Whole blood tGSH.	6
Pastural *et al*. 2009 [82] (Canada)	15 cases, 12 controls	Cases 7.9 y (2-13 y) 100%M Controls 8.7 y (4-17 y) 75%M	Enrolled by Jonty Foundation	9/12 siblings 3/12 community controls with no family history of autism, age not gender matched.	DSM-IV for autistic disorder.	Plasma homocysteine, cysteine and tGSH. Glutamate toxicity in neuronal, astrocyte and hepatocyte cell cultures.	2
Mostafa *et al*. 2010 [83] (Egypt)	44 cases, 44 controls	Cases median 8 y (3.5-12 y), 68%M Controls median 8 y (4-12 y),	Patients attending the Pediatric Neuro-Psychiatric Clinic, Children's Hospital, Ain Shams University	Siblings of children with minor illnesses attending the Out- patients' Clinic, Children's Hospital, Ain Shams University.	DSM-IV for autistic disorder.	Plasma GPx activity	7
Vergani *et al*. 2011 [84] (Italy)	28 cases, 32 controls	Cases 2-6 y, 75%M Controls 62.5%M	Not stated.	Not stated.	DSM-IV for autistic disorder.	Erythrocyte GPx activity.	3
Al-Yafee *et* *al*. 2011 [85] (Saudi Arabia)	20 cases 20 controls	Cases (3-16 y), 100%M, 100% IQ < 80. Controls 3-16 y, 100% M.	Autism Research and Treatment Centre clinic.	Well Baby Clinic, King Khaled University Hospital.	ADI-R and ADOS and Developmental, dimensional diagnostic interview.	Total glutathione, oxidised glutathione, tGSH:GSSG, glutathione reductase and gluthathione-s-transferase activity.	5
Tirouvanziam *et al. *2011 [86] (USA)	27 cases 20 controls	Cases 7.0 ± 2.3 y, 77.8%M Controls 7.3 ± 2.5 y, 45%M	Community referrals.	Community advertisements.	ADI-R and ADOS for autistic disorder. ADI-R score 7-10 plus ADOS criteria for autism for PDD-NOS.	Platelet poor plasma glutamine, serine and glycine.	5
Adams *et al*. 2011 [87] (USA)	55 cases 44 controls	Cases 10.0 ± 3.1 y, 89%M Controls 11.0 ± 3.1 y, 89%M	Autism Society of Greater Phoenix and Arizona Division of Developmental Disorders.	Autism Society of Greater Phoenix and Arizona Division of Developmental Disorders.	Prior diagnosis by a child psychiatrist or developmental paediatrician.	Plasma glutamate, serine, GSH and GSSG. Selenium (whole blood and erythrocyte).	6

***Case Control Level III-3***							
Aldred *et al*. 2003 [88] (UK)	23 cases (12 autistic disorder, 11 Asperger's syndrome), 32 parents, 23 siblings.	Cases Autism 16.4 ± 9.04 y, 92%M Aspergers 15.7 ± 8.63 y, 91%M Parents 44.1 ± 6.9 y, 44%M Siblings 16.5 ± 6.4 y, 39%M	Child psychiatric out-patient clinics in Dublin and Tipperary.	Children admitted to Birmingham Children's Hospital for trauma or minor elective surgery.	DSM-IV for autistic disorder or Asperger's Syndrome.	Plasma serine and glycine.	5
Arnold *et al*. 2003 [89]	36 cases (mixed autistic disorder and PDD-NOS) (10 gluten/casein free diet). 24 controls.	Cases Aged < 5 years, gender unknown. Controls Age and gender matched with cases on a regular diet.	Retrospective medical records from Kirsch Developmental Services Center or the Genetic Consultation Clinic at the University of Rochester School of Medicine & Dentistry 1996-1998.	Children with developmental delay not autism. Source not stated.	DSM-IV supported by CARS or the pervasive Developmental Disorders Screening Test.	Plasma glutamine.	5
Geier & Geier 2009 [90] (USA)	28 cases (20 autistic disorder, 8 other ASD) 64 controls for cysteine and 120 controls for glutathione measurements.	Cases 5.8 ± 2.7 y, 82% M 50% mild, 50% severe autism. Controls 2-16 y. Gender unknown.	Dallas/Fort Worth, Texas area.	Prospective samples from non- autistic children aged 2-16 y collected by the participating laboratory.	CARS > 30. Mild autism CARS 30-38.5 Severe autism CARS > 38.5	Cysteine, reduced and oxidised glutathione.	3
Geier & Geier 2009 [91] (USA)	38 cases (28 10 other ASD) Controls as above.	Cases 6.0 ± 2.6 y, 89.5% M Controls as above.	As above.	As above	CARS > 30	As above.	3
Sankar 1979 [92] (USA)	19 cases	Age within the range 5-16 y, 100%M Medication and supplement free for 3 weeks prior to fasted blood draw.	Children admitted to Creedmoor State Hospital	Used reference ranges from literature.	Onset from infancy with severe emotion-al isolation; failure to relate to objects & persons; failure to develop speech & communication. If speech present, it is a non-communicative type. Stereotypy of motor behaviour.	Serum vitamin B_6_.	4
Khaludeenin & Philpott 1980 [93] (USA)	9 cases	Cases mean 9 y, 78%M	Consecutive cases at Philpott clinic	Used reference range from literature.	Not provided	Plasma cystine, cystathione, glutamic acid and vitamin B_6_.	

***Level IV (Case series)***							
Geier & Geier 2006 [66] (USA)	16 cases	Cases 5.9 ± 2.1 y	Consecutive pre-pubertal age children (≤ 11 y) with a previous diagnosis of regressive autism or PDD-NOS presenting as an outpatient at the Genetic Centres of America between Nov. 2004 and Nov. 2005.	Reference range provided by participating laboratories.	Not provided, relied on previous diagnosis.	Plasma cysteine and reduced glutathione, serum cystathionine and cysteine.	

***Level IV (Case report)***							
Moretti *et al*. 2005 [54] (USA)	1 case	Case aged 6 y, F	Not stated.	n/a	ADOS, ADI-R for autistic disorder.	Cerebral spinal fluid homocysteine.	
***Genetic studies***							
Bowers *et al*. 2011 [94] (USA)	318 families (1,149 individuals including 457 children) plus 3,327 participants from independent AGRE families for replication.	Cases 77.6% M, 321 sibships, Caucasian 91.68%, American black 0.22%, unknown 8.1%.	Autism Genetic Resource Exchange.	Family members of cases from the Autism Genetic Resource Exchange.	ADI-R and ADOS for autism spectrum disorders.	308 SNPs of 42 candidate genes related to glutathione.	4

Ming *et a*l. 2010 [95] (USA)	103 cases, (101 autistic disorder, 2 PDD-NOS) including 68 case trios.	Cases 86%M	New Jersey Center for Outreach & Services for the Autism Community and Department of Pediatrics UMDNJ.	Not relevant.	ADI-R and ADOS-G for autistic disorder.	GPx-1 polymorphisms.	3

Rose *et al*. 2008 [50] (USA)	451 cases, 251 controls	Cases aged 6.9 ± 2.9 y (3-14 y) Controls aged 10.8 ± 4.1. Gender not stated.	Participants referred by the Dennis Developmental Center at the University of Arkansas for Medical Sciences and from clinics of participating physicians in New York and Florida.	Glutathione values from controls in James 2006.	DSM-IV and ADOS or CARS.	tGSH, fGSH, GSSG stratified by ALAD polymorphism.	4

Buyske *et al*. 2006 [96] USA)	54 case parent trios 172 controls	45 cases diagnosed as autistic disorder using both instruments, 9 diagnosed as autistic disorder using one instrument and PDD- NOS using the other.	New Jersey Center for Outreach Services for the Autism Community and the Dept of Pediatrics, UMDNJ -Robert Wood Johnson Medical School.	UMDNJ clinics and individuals married into dominant pedigrees of other disorders.	ADI-R and ADOS-G for autistic disorder.	GST-M1 polymorphisms	4

James *et al*. 2006 [47] (USA)	360 cases 205 controls (comprising 73 children and 132 adult females).	Cases 7.3 ± 3.2 y (3-14 y), 89% M Controls 10.8 ± 4.1 y, gender not stated (n = 73) plus adult females of child bearing age (n = 132).	Participants referred from autism clinics of participating physicians in New York and Florida.	Participants of studies of Down syndrome, cystic fibrosis and a congenital heart failure risk study.	DSM-IV, ADOS or CARS for autistic disorder.	GST-M1 and T1 polymorphisms.	3

Serajee *et al*. 2004 [97] (USA)	196 case parent trios (581 individuals).	Not stated.	Autism Genetic Resource Exchange. Random selection of one affected sibling from each multiplex family.	Not relevant.	DSM-IV for autism spectrum disorder.	GST-P1 polymorphisms.	6

^1^Unless otherwise stated, children with epilepsy, genetic, mental health or metabolic conditions were excluded.

DSM Diagnostic and Statistical Manual of Mental Disorders CARS Childhood Autism Rating Scale

ADI-R Autism Diagnostic Interview (Revised) ADOS Autism Diagnostic Observation Schedule

ASD Autism Spectrum Disorder PDD-NOS Pervasive developmental disorder -not otherwise specified

AGRE Autism Genetic Resource Exchange UMDNJ University of Medicine and Dentistry of New Jersey

ALAD delta-amino levulinic acid dehydratase GPx-1 Glutathione peroxidase

SHMT1 Serine hydroxyl methyl transferase GST-P1 Glutathione-s-transferase Pi 1

tGSH Total glutathione GSSG Oxidised glutathione

GST Glutathione-S-transferase HBOT Hyperbaric oxygen therapy

* Based on baseline data for cases and controls

An assessment of study quality is presented in Tables 4, 5, 6 and 7. The case definition used to include participants in the studies varied over time. The case definition for autistic disorder was not standardised until 1980 when it was included in the DSM-III. Asperger's Syndrome and PDD-NOS were added to the DSM-IV in 1994 which broadened the definition to include many children who were previously undiagnosed. While early studies centred on cases obtained from institutionalised psychiatric settings [92,93,98], cases were later recruited through internal research registers [64], multiple centres [47,50,64,70,71,88,95,96] or community advertisements [75]. Although diagnosis was independently confirmed in several studies [59,60,69,70,72,75,83,85,91,92], most relied on medical records or parent reports. None of the studies had used a structured sampling frame for case ascertainment making them prone to selection bias. Information about case ascertainment was not provided for eight studies [48,49,54,59,74,78,81,84].

**Table 4:** Scores for assessment of quality for case control studies using the Newcastle Ottawa Scale

	Golse **et al**. 1978	Sankar **et al**. 1979	Khaleel uddin & Philpot 1980	Rolf et al. 1993	Visconti **et al**. 1994	D'Eufe **mia et al**. 1995	Yorbik et al. 2002	Sogut et al. 2003	Aldred et al. 2003	James et al. 2004	Adams et al. 2004	James et al. 2006	Pasca et al. 2006	Shinohe et al. 2006	Adams et al. 2006	Adams et al. 2007	Rossign **ol et al**. 2007	Suh et al.2008
1. Selection																		
Case definition	0	2	0	1	2	2	1	2	1	1	1	1	1	2	1	2	1	1
Representativeness	0	0	0	0	0	0	0	0	0	0	0	0	0	0	0	0	0	0
Selection of controls	0	0	1	0	0	0	1	1	1	0	0	0	0	2	0	0	0	1
Definition of controls	0	0	1	1	1	2	1	1	1	1	0	0	1	2	0	1	0	2
2. Comparability																		

Control for confounding	0	0	0	1	0	0	0	0	1	0	2	0	0	2	0	0	0	0

3. Exposure																		
Ascertainment	0	1	0	0	0	0	0	0	0	0	0	0	0	0	1	0	0	0
Method same for cases/controls	1	1	1	1	1	1	1	1	1	1	1	1	1	1	1	1	1	1

**Overall score**	**1**	**4**	**3**	**4**	**4**	**5**	**4**	**5**	**5**	**3**	**4**	**2**	**2**	**9**	**3**	**4**	**2**	**5**

**Table 5:** Scores for assessment of quality for case control studies using the Newcastle Ottawa Scale

	Jory & McGinnis 2008	Rose et al. 2008	Vojdani et al. 2008	James et al. 2009	James et al. 2009 *(in vitro)*	Al-Gadani et al. 2009	Pasca et al. 2009	Pastural et al. 2009	Geier & Geier 2009	Adams et al. 2009	Mostafa et al. 2010	Vergani et al. 2011	Al-Yafee et al. 2011	Adams et al. 2011	Tirouvanzium et al. 2011	Max score
1 Selection																
Case definition	1	1	1	1	2	2	2	1	2	1	2	1	2	1	1	**2**
Representativeness	0	0	0	0	0	0	0	0	0	0	0	0	0	0	0	**1**
Selection of controls	0	0	1	0	0	0	0	0	0	0	1	0	1	0	0	**2**
Definition of controls	0	0	0	1	1	0	1	0	0	0	1	1	1	2	1	**2**
2 Comparability																
Control for confounding	0	0	0	1	1	1	2	0	0	0	2	0	0	0	2	**2**
3 Exposure																
Ascertainment	0	0	0	0	0	0	0	0	0	1	0	0	0	1	0	**1**
Method same for cases/controls	1	1	1	1	1	1	1	1	1	1	1	1	1	1	1	**1**
**Overall score**	**2**	**2**	**3**	**4**	**5**	**4**	**6**	**2**	**3**	**3**	**7**	**3**	**5**	**5**	**5**	**9**

**Table 6:** Scores for assessment of quality for genetic studies the Newcastle Ottawa Scale

	Bowers et al. 2011	Ming et al. 2010	Buyske et al. 2006	James et al. 2006	Rose et al. 2006	Serajee 2004	Max Score
1 Selection							

Case definition	2	2	1	1	1	2	**2**

Representativeness	0	0	0	0	0	0	**1**

Selection of controls	trios	trios	trios	0	0	trios	**2**

Definition of controls	n/a	n/a	n/a	0	0	n/a	**2**

2 Comparability							

Control for confounding	n/a	n/a	n/a	0	0	n/a	**n/a**

3 Exposure							

Ascertainment	0	0	0	0	0	0	**1**

Method same cases/controls	1	1	1	1	1	1	**1**

4 Genetics							

HW equilibrium	1	0	1	1	1	1	**1**

Power calculations	n/a	0	1	0	0	1	**1**

Control multiple comparisons	0	0	0	0	1	1	**1**

Population stratification	n/a	n/a	0	0	0	n/a	**1**

**Overall score**	**4**	**3**	**4**	**3**	**4**	**6**	**13**

**Table 7:** Scores for assessment of risk of bias for intervention studies

Risk of bias	Bertoglio et al. 2011	James et al. 2009	Adams et al. 2009	Rossignol et al. 2007	James et al. 2004
Randomisation	Uncertain	Open label	Uncertain	Open label	Open label

Concealment of allocation	Low risk	Open label	Low risk	Open label	Open label

Blinding	Low risk	Open label	Low risk	Open label	Open label

Incomplete outcome data	High risk	Low risk	High risk	Low risk	Low risk

Selective reporting	High risk	Low risk	High risk	Low risk	Low risk

**Overall score**	5	4	5	4	4

Scoring

Low risk = 2; Uncertain = 1; High risk/open label = 0

Ascertainment and definition of controls also varied widely. While two studies sourced their controls by community advertising [75,86], most were sourced from hospitals, clinics or schools [71,72,77,79,83,85,88,92,93] and fourteen studies did not provide information on the source of their controls [48,59,60,63,68-70,73,74,76,78,81,84]. With respect to definition of controls, most studies recruited healthy children with no information about family history of autism spectrum disorders, although, four studies did ensure that controls did not have either a family history or sibling with autism [70,75,77,87] and one screened for autism traits [59]. At the other end of the scale, controls for four studies were poorly defined potentially biasing the results [46,47,89,98]. Control values from one of these studies [47] were used for two later studies [49,50]. Additionally, three studies relied on laboratory reference ranges [65,92,93].

Gender is a potential confounder in studies of autistic disorder because the condition is four times more common in males than females [99]. Only five studies were gender matched [60,73,75,83,89], four did not provide the gender of cases or controls [50,59,71,89] and nine provided the gender of cases but not controls [46-48,50,70,73,79,83,91]. Age may also be a potential confounder as serum glutamate was elevated in adults with autistic disorder compared to adult controls [75] but was not significantly different in children with autistic disorder compared to child controls [69,70]. In contrast, serum glycine and serine were not significantly different in either adults [75] or children [69,70] when levels in autistic disorder were compared to controls. One study included a range of participants from childhood to early adulthood, however, the findings were not stratified according to age [88].

All studies included in the review treated cases and controls equally. Laboratory blinding as to case and control status occurred for only one research group [65,76,87], although others were blinded to case status but not controls, for example, where the laboratory provided the control data [88,90,91] or reference ranges [65,67,92,93] or where another study was used for controls [49,50]. Most studies did not state whether the laboratory was blinded.

Genetic studies were assessed for quality using the Newcastle Ottawa Scale plus additional criteria that included consideration of Hardy Weinberg equilibrium, power of the study, population stratification and correction for multiple comparisons. All except one of the six genetic studies considered Hardy Weinberg equilibrium [47,50,94,96,97], two provided power calculations [96,97] and two adjusted for multiple comparisons [50,97] (although a footnote indicating that the associations were no longer statistically significant was not added in one case) [50]. While population stratification is not relevant for transmission linkage studies [94-97], neither of the remaining studies were adjusted for this [47,50].

Both of the double blinded randomised intervention trials provided information about concealment and the laboratory was blinded thereby reducing performance and detection bias [64,65]. Neither provided information about the randomisation process, complete outcome data and full reporting of results. While Bertoglio *et al. *2010 state that 30 children completed the 12-week trial, closer inspection of the paper suggests that at least 32 children started the trial (see Table 1 in Bertoglio *et al. *2010), however, no information on dropout or loss to follow-up was provided. Furthermore outcome data was only provided for the 'responder' sub-group in a form that was difficult to interpret. Adams *et al. *2009 randomised children to receive either topical glutathione or a placebo before being given one round of a chelating agent with erythrocyte glutathione tested at baseline and 1-2 months following the intervention [65]. It is not clear whether it is a typographical error, however, Table 1 of the study states that 77 children participated in the first phase of the study, but baseline data for erythrocyte glutathione is only provided for 72 children. Although the paper states that 49 started the second phase of the study and therefore, according to the protocol, had a second glutathione measurement, pre- and post-intervention erythrocyte glutathione is only provided for 38 participants with no comparison between the two arms of the study with levels being compared to an adult reference range provided by the laboratory. The second phase of the study involved 'high excreters' of urinary metal ions being given a further 6 rounds of chelation if allocated to the topical glutathione arm or 6 rounds of placebo if previously allocated to the topical placebo arm of the study. Erythrocyte glutathione was not measured at the completion of the second phase of the study.

The open-label study design used in the remaining three intervention studies left them at high risk of selection, performance and detection bias [46,49,65], however, all studies provided complete outcome data and full reporting of results.

A kappa score of 0.87 was obtained which indicates a high level of agreement between raters for the assessment of quality of articles.

### *In vitro *studies of the γ-glutamyl cycle

Table 8 summarises the findings of an *in vitro *study of γ-glutamyl cycle metabolites [80]. Decreased free glutathione (fGSH) and increased GSSG were observed in both cytosol and mitochondrial extracts obtained from lymphoblastoid cell lines derived from children with autistic disorder compared to unaffected controls resulting in a decreased GSH:GSSG. Exposure to physiological levels of nitrosative stress showed no difference in the magnitude of GSH:GSSG from cells derived from children with autistic disorder compared to healthy controls, however, the baseline GSH:GSSG was significantly lower (by 30%) in cells from children with autistic disorder.

**Table 8:** *In vitro *studies of γ-glutamyl cycle metabolites

Model	Metabolite	Study	**Cases**^1^	**Controls**^**1**^	*P *values	**Overall finding**^**2**^	Comments
Lymphoblastoid cell lines	Free glutathione	James *et al. *2009 [80]	21.72 ± 4.3	26.48 ± 3.5	0.021	lower	Whole cell etract - intracellular glutathione

			1.75 ± 0.3	2.64 ± 0.7	0.001	lower	Mitochondria - intracellular glutathione

			19.8 ± 4.1	23.5 ± 4.5	< 0.04	lower	Whole cell extract - without nitrosative stress

			17.4 ± 3.9	18.3 ± 4.1			Whole cell extract - with nitrosative stress

	Oxidised glutathione	James *et al. *2009 [80]	0.36 ± 0.06	0.29 ± 0.07	< 0.001	higher	Whole cell extract - intracellular glutathione

			0.37 ± 0.11	0.26 ± 0.12	0.059	higher	Mitochondria - intracellular glutathione

			0.26 ± 0.08	0.19 ± 0.04	< 0.04	higher	Whole cell extract - without nitrosative stress

			0.51 ± 0.35	0.48 ± 0.30			Whole cell extract - with nitrosative stress

	Free: oxidised glutathione	James *et al. *2009 [80]	61.81 ± 10.6	99.14 ± 33.5	< 0.001	lower	Whole cell extract - intracellular glutathione

			5.06 ± 1.3	11.63 ± 3.9	< 0.001	lower	Mitochondria - intracellular glutathione

			19.8 ± 4.1	23.5 ± 4.5	< 0.04	lower	Whole cell extract - without nitrosative stress

			17.4 ± 3.9	18.3 ± 4.1			Whole cell extract - with nitrosative stress

^1 ^mean ± standard deviation, nmol/mg protein

^2 ^cases relative to controls

### Metabolites and cofactors of the γ-glutamyl cycle and trans-sulphuration pathway

**Figure 4:**
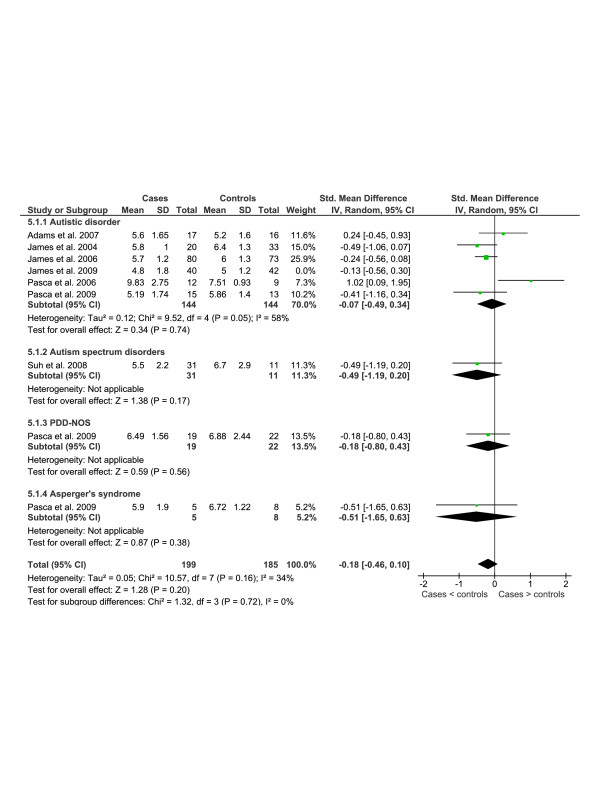
**Meta-analysis of studies that compared plasma homocysteine in children with autism spectrum disorders to healthy controls**.

**Figure 5:**
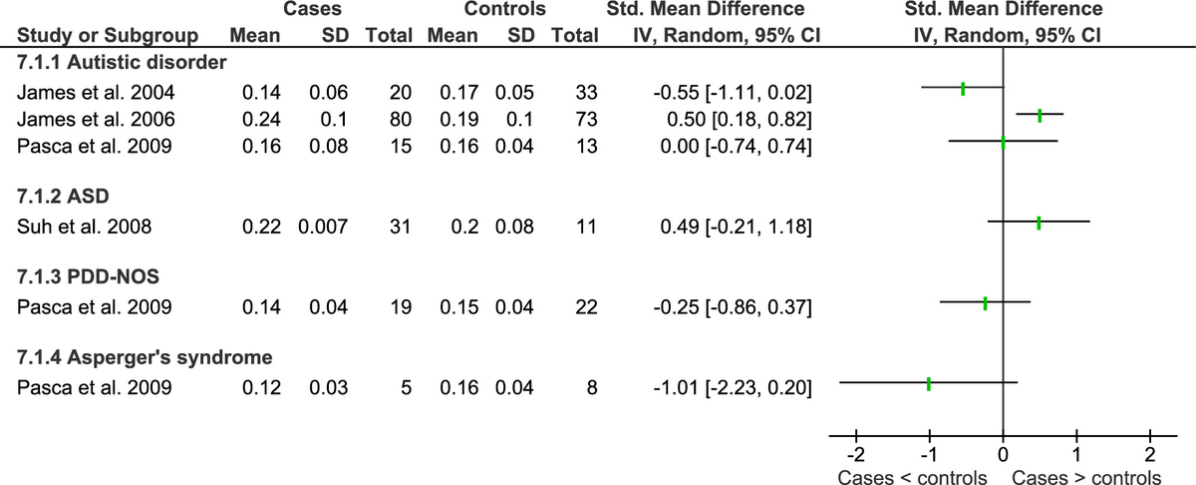
**Heterogeneity of studies that compared plasma cystathione in children with autistic spectrum disorder and healthy controls**.

**Figure 6:**
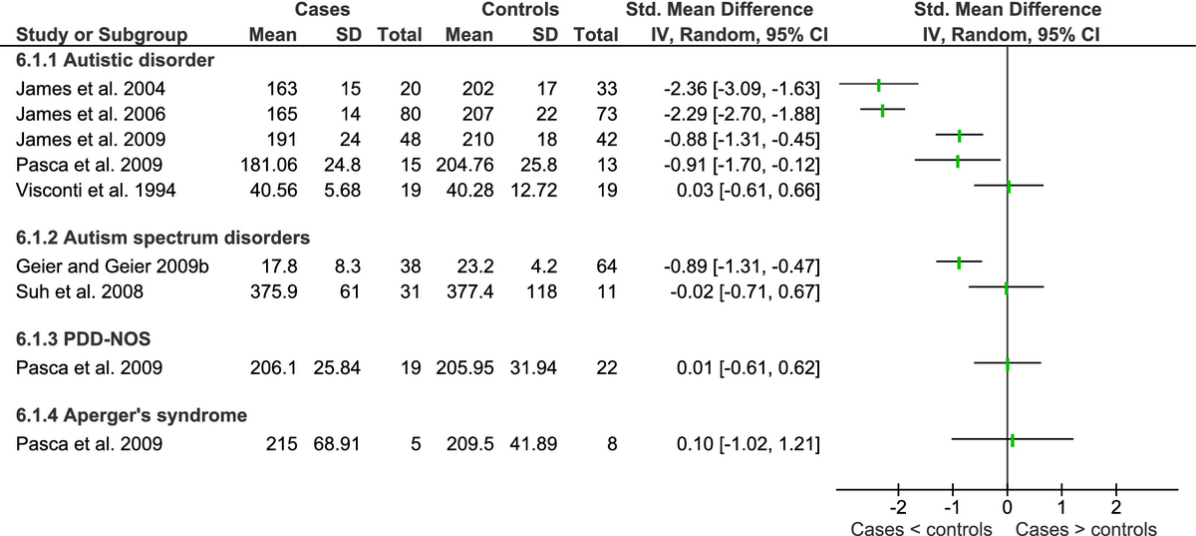
**Heterogeneity of studies that compared plasma cysteine in children with autistic spectrum disorder and healthy controls**.

Data from key studies of metabolites of the γ-glutamyl cycle and trans-sulphuration pathway is shown in Figures 4, 5 and 6 and a summary of additional studies presented in Table 9.

**Table 9:** *In vivo *studies examining an association between metabolites of the γ-glutamyl cycle or trans-sulfation pathway and autism spectrum disorders

		Study	**Cases**^**1**^	**Controls**^**1**^	*P *values	Overall finding	Comments
***Total glutathione (μmol/L)***							

Plasma	AD	Rose *et al. *2008 [50]	5.69 ± 1.3^7^ 5.08 ± 1.3^8^	7.53 ± 1.7	< 0.001	lower	ALAD CG + CC significantly lower than GG *p *= 0.007.

		Pastural *et al. *2009 [82]	Not provided	Not provided	< 0.05	lower	

Erythrocyte	ASD	Adams *et al. *2009 [65,67]	501 ± 246	427-714		same	Paediatric values are lower than the reference range for adults provided by the laboratory.

Whole	AD	Pasca *et al. *2009^4 ^[60]	161.16 ± 41.36	242.67 ± 118.77	0.02	lower	SEM converted to SD. Not treated to prevent oxidation

Blood	PDD-NOS	Pasca *et al. *2009^4 ^[60]	209.96 ± 94.63	213.32 ± 98.55	ns	same	As above.

	AS	Pasca *et al. *2009^4 ^[60]	147.31 ± 74.26	171.29 ± 92.69	ns	same	As above.

***Free glutathione (μmol/L)***							

Plasma	AD	James *et al. *2006^3,4 ^[47]	1.4 ± 0.5	2.2 ± 0.9	< 0.0001	lower	

		Rose *et al. *2008 [50]	1.60 ± 0.5^7^	2.2 ± 0.9	< 0.001	lower	ALAD CG + CC significantly lower than GG *P *= 0.02
			1.4 ± 0.4^8^				Same controls as James *et al. *2006.

		James *et al. *2009^3,4 ^[48]	1.5 ± 0.4	2.8 ± 0.8	0.008	lower	Cases with abnormal methylation or tGSH:GSSG only.

***Reduced glutathione (μmol/L)***							

Plasma	ASD	Geier & Geier 2006 [66]	64% lower		< 0.01	lower	Sample preparation not provided.

		Geier & Geier 2009 [90]	3.1 ± 0.53	4.2 ± 0.72	< 0.0001	lower	

	ASD	Geier & Geier 2009 [91]	3.14 ± 0.56	4.2 ± 0.72	< 0.0001	lower	

		Adams *et al. *2011 [87]	3.23 ± 0.48	4.09 ± 0.79	< 0.0001	lower	

***Oxidised glutathione (μmol/L)***							

Plasma	AD	Rose *et al. *2008 [50]	0.32 ± 0.12^7^ 0.32 ± 0.12^8^	0.24 ± 0.1	< 0.001	higher	No significant difference between ALAD CG + CC and GG.

***Total glutathione: oxidised glutathione***							

Plasma	AD	James *et al. *2004^3 ^[46]	8.6 ± 3.5	25.5 ± 8.9	< 0.001	lower	

		James *et al. *2006^3,4 ^[47]	14.7 ± 6.2	28.2 ± 7.0	< 0.001	lower	

		Rossignol *et al. *2007^5 ^[49]	28.47 ± 4.59^9^	28.2 ± 7.0			Cases more likely to be severe as requested
			44.68 ± 14.19^10^				HBOT. Controls from James et al. 2006.

		Rose *et al. *2008 [50]	20.45 ± 8.7^7^	28.2 ± 7.0	< 0.001	lower	ALAD CG + CC significantly lower than GG
			17.4 ± 5.7^8^				*P *= 0.03. Controls from James et al. 2006.

		James *et al. *2009^3,4 ^[48]	21 ± 6	47 ± 18	< 0.005	lower	Cases abnormal methylation or tGSH:GSSG only.

***Free: oxidised glutathione***							

Plasma	AD	James *et al. *2006^3,4 ^[47]	4.9 ± 2.2	7.9 ± 3.5	< 0.0001	lower	

		Rose *et al. *2008 [50]	5.64 ± 2.6^7^ 4.81 ± 1.8^8^	7.9 ± 3.5	< 0.001	lower	ALAD CG + CC significantly lower than GG *P *= 0.05

***Reduced: oxidised glutathione***							

Plasma	ASD	Adams *et al. *2011 [87]	8.0 ± 3.2	10.1 ± 4.5	0.01	lower	

		Al-Yafee *et al. *2011 [85]	8.03 ± 2.46	26.07 ± 5.03	0.001	lower	

***Homocysteine (μmol/L)***							

Plasma	AD	Moretti *et al. *2005 [54]	6	4-14		ns	Case study compared with reference values

		Pastural *et al. *2009 [82]	Not provided	Not provided	ns	ns	Result control normalised graph.

Serum		Geier & Geier 2006 [66]	62% lower		< 0.01		Does not state whether fasted or not.

CSF	AD	Moretti *et al. *2005 [54]	0.424	0.032-0.114		high	Case study compared with reference values

***Cysteine (μmol/L)***							

Plasma	AD	Pastural *et al. *2009 [82]	Not provided	Not provided	< 0.05	lower	Result provided graphically as relative to homocysteine.

	ASD	Geier & Geier 2006 [66]	81% lower		< 0.01		

Serum	AD	Visconti *et al. *1994^6 ^[69]	40.56 ± 5.68^11^ 41.04 ± 15.56^12^	40.26 ± 12.72	ns	same	

***Cystine (mmol/L)***							

Plasma	AD	Khaleeluddin & Philpott 1980 [93]	5/6 low				Concentrations and reference range not provided.

		D'Eufemia *et al. *1995^3 ^[70]	78.3 ± 17.5	72.5 ± 19.9	ns	ns	Units incorrectly labelled mmol/L in Table 1. Correctly labelled μmol/L in Figure 3.

	ASD	Adams *et al. *2011 [87]	32.2 ± 8.2	34.8 ± 7.4	ns	ns	Fasted.

***Cystathione (μmol/L)***							

Plasma	AD	Khaleeluddin & Philpott 1980 [93]	1/6 high				

Serum	ASD	Geier & Geier 2006 [66]	68% low		< 0.05		Details of sample preparation not provided.

***Cysteinylglycine (μmol/L)***							

Plasma	AD	James *et al. *2006^4,5 ^[47]	38.9 ± 11	39.4 ± 7.3	0.78	ns	

		James *et al. *2009^4,5 ^[48]	40 ± 9	45 ± 6	< 0.005	lower	Cases abnormal methylation or tGSH:GSSG only.

	ASD	Suh *et al. *2008 [77]	17.5 ± 6.8	10.5 ± 4.1	0.0008	higher	

***Serine (μmol/L)***							

Serum	AD	Visconti *et al. *1994^6 ^[69]	130.59 ± 24.84^9^	143.79 ± 30.08	ns	ns	
			151.45 ± 50.43^10^	143.79 ± 30.08	ns	ns	

		D'Eufemia *et al. *1995^3 ^[70]	163.5 ± 32.1	169.1 ± 47.3	ns	ns	Units incorrectly labelled mmol/L in Table 1. Correctly labelled μmol/L in Figure 3.

		Shinohe *et al. *2006^4 ^[75]			ns	ns	Results presented graphically

Plasma	AD	Pasca *et al. *2009^5 ^[60]	99.46 ± 13.56	125.23 ± 47.31	0.08	trend	SEM converted to SD.

	ASD	Aldred *et al. *2003 [88]			ns	ns	Results presented graphically.

		Adams et al. 2011 [87]	104 ± 25	94.7 ± 21	0.04	high	

(platelet poor)		Tirouvanziam *et al. *2011 [86]	85.23 ± 26.5	112.30 ± 27.3	0.0013	low	Data obtained from author.

	PDD-NOS	Pasca *et al. *2009^5 ^[60]	113.31 ± 22.84	114.6 ± 38.23	ns	ns	SEM converted to SD.

	AS	Pasca *et al. *2009^5 ^[60]	96.2 ± 14.3	124.75 ± 49.07	ns	ns	SEM converted to SD.

***Glycine (μmol/L)***							

Serum	AD	Visconti *et al. *1994^6 ^[69]	225.88 ± 36.23^8^	245.63 ± 60.19	ns	ns	

			225.06 ± 24.07^9^		ns	ns	

		D'Eufemia *et al. *1995^3 ^[70]	246.7 ± 52.2	257.7 ± 55.3	ns	ns	Units incorrectly labelled mmol/L in Table 1. Correctly labelled μmol/L in figures.

		Shinohe *et al. *2006^4 ^[75]			ns	ns	Results presented graphically

Plasma	AD	Pasca *et al. *2009 [60]	184.20 ± 46.67	217.23 ± 52.46	0.09	ns	SEM converted to SD

	ASD	Aldred *et al. *2003 [88]			ns	ns	Results presented graphically.

		Adams *et al. *2011 [87]	267 ± 81	273 ± 101	ns	ns	

(platelet poor)		Tirouvanziam *et al. *2011 [86]	192.8 ± 46.8	190.3 ± 49.5	ns	ns	Data obtained from author.

	PDD-NOS	Pasca *et al. *2009^5 ^[60]	207.94 ± 10.31	209.73 ± 42.31	ns	ns	SEM converted to SD.

	AS	Pasca *et al. *2009^5 ^[60]	188.6 ± 30.39	224.0 ± 49.81	ns	ns	SEM converted to SD.

***Glutamate (μmol/L)***							

Serum	AD	Visconti *et al. *1994^6 ^[69]	61.89 ± 22.69^8^	77.16 ± 50.01	ns	ns	
			72.28 ± 44.42^9^	77.16 ± 50.01	ns	ns	

		D'Eufemia *et al. *1995^3 ^[70]	77.3 ± 24.5	72.4 ± 21.2	ns	ns	Units incorrectly labelled mmol/L in Table 1. Correctly labelled μmol/L in figures.

		Shinohe *et al. *2006^4 ^[75]	89.2 ± 21.5	61.1 ± 16.5	< 0.001	higher	High correlation with ADI-R social scores (r = 0.523, *P *= 0.026)

	ASD	Adams *et al. *2011 [87]	65 ± 15	55 ± 13	0.001	higher	

Plasma		Arnold *et al. *2003 [89]	51 ± 32^13^	48 ± 15		ns	
			42 ± 23^14^	48 ± 15		ns	

Plasma (platelet poor)		Tirouvanziam *et al. *2011 [86]	104.06 ± 33.85	82.71 ± 34.20	0.039	higher	Data obtained from authors.

***Glutamate (nmol/10^8^)***							

Platelets	AD	Rolf *et al. *1993^4 ^[68]	4.8 ± 1.2	6.0 ± 1.2	< 0.02	lower	Findings presented graphically.

***Vitamin B6 (nmol/L)***							

Plasma	AD	Sankar 1979^7 ^[92]	753.6 ± 31.7	Reference	Not	higher	
				Range (147.8-254.2)	provided		

		Khaleeluddin & Philpott	4/9 high		Not	higher	

		1980 [93]			provided	higher	

	ASD	Adams *et al. *2004 [73]	224.55 ± 30.35	129.47	< 0.001	higher	

		Adams *et al. *2006 [76]	226.58 ± 84.97	145.66 ± 35.60	0.001	higher	

***Vitamin B6 as pyridoxyl-5-phosphate (nmol/L)***							

Erythrocyte	ASD	Adams *et al. *2011 [87]	72.44 ± 64.75	61.51 ± 21.45	ns	ns	

***Selenium (μmol/L)***							

Erythocytes	AD	Jory and McGinnis 2007^4,5 ^[78]	3.12 ± 0.54	3.67 ± 0.38	0.0006	lower	

Whole blood	ASD	Adams *et al. *2011 [87]	2.63 ± 0.36	2.67 ± 0.25	ns	ns	

***Selenium (μg/g)***							

Erythrocytes	ASD	Adams *et al. *2011 [87]	0.24 ± 0.04	0.23 ± 0.03	ns	ns	

^1^Mean ± standard deviation ^2 ^Cases relative to controls ^3 ^Free from relevant medications ^4 ^Free from supplementation^ 5 ^Most on folinic acid or methyl-cobalamin ^6^One case, one control on thioridazine ^7 ^ALAD GG polymorphism ^8 ^ALAD CG + CC polymorphism ^9^Prior to 40 sessions HBOT at 1.3 atm ^10^Prior to 40 sessions HBOT at 1.5 atm ^11 ^Normal EEG ^12^Abnormal EEG^ 13 ^Normal diet ^14^Gluten/caseine free diet ns not significant AD Autistic disorder

ASD Autism spectrum disorder PDD-NOS Pervasive developmental disorder- not otherwise specified. GSH Reduced glutathione GSSG Oxidised glutathione tGSH Total glutathione ALAD Delta aminolevulinic acid dehydratase HBOT Hyperbaric oxygen therapy SEM Standard error of the mean SD standard deviation

The largest and most comprehensive study to date provided data for multiple metabolites of the γ-glutamyl cycle and trans-sulphuration pathway [47]. This study reported significantly lower levels of GSH (by 32%) and higher levels of GSSG (by 66%) in plasma of children with autistic disorder compared to controls, together with significantly lower homocysteine and cysteine levels, while cystathione levels were significantly higher and cysteinyl-glycine levels were not significantly different [47]. These findings confirm those of an earlier pilot study by the same researchers with the exception that cystathione was found to be lower in children with autistic disorder in the pilot study [46], as well as a later study by the same research group which focussed on a subgroup of children with autistic disorder who had abnormal methylation and/or GSH:GSSG [48].

Plasma homocysteine levels for the above studies [46-48] showed that there was no statistically significant difference between children with autistic disorder and controls which has been replicated by a number of other research groups for children with autistic disorder [59,60,82], PDD-NOS and Asperger's syndrome [60] as well as a mixed sample of children with autism spectrum disorders [77]. The only study to report a significant increase in plasma homocysteine in children with autistic disorder [74] was not replicated by the same research group using a fasted sample [60]. Examination of statistical heterogeneity showed low heterogeneity overall (I^2 ^= 34%) and no heterogeneity between diagnostic subgroups (I^2 ^= 0%) (Figure 4). Meta-analysis resulted in a standardised mean difference (SMD) of -0.18 (95%CI -0.46-0.10) across 199 cases and 185 controls using a random effects model. Data from James *et al. *2009 was not included in the analysis because the cases were selected for low methylation ratio or GSH:GSSG, however, the data is presented in Figure 4.

Similarly, no significant difference was observed in plasma cystathione from children with autistic disorder, PDD-NOS, Asperger's Syndrome or mixed autism spectrum disorders [46,60,77], although another study report it to be significantly higher [47] in children with autistic disorder than controls (Figure 5). Examination of statistical heterogeneity showed that there was substantial overall heterogeneity (I^2 ^= 70%) with moderate heterogeneity between diagnostic subgroups (I^2 ^= 41.4%). It is hard to explain the heterogeneity given that two of the larger studies were conducted by the same research group using the same methodology [46,47].

Serine is required for synthesis of cystathione from homocysteine. Four studies found no significant difference in serum or plasma serine levels between children and adults with or without autistic disorder, PDD-NOS, Asperger's Syndrome or autism spectrum disorders (mixed sample) [60,69,70,75], one study showed a trend towards a decrease in children with autistic disorder [60] and another reported significantly increased plasma serine in children with autism spectrum disorders [87] and significantly lower levels of serine were reported for platelet poor plasma in autism spectrum disorders [86]. Factors that may have contributed to the heterogeneity between studies include fasting status, differing laboratory methods and varied selection of controls as well as correction for multiple comparisons.

Studies showing that plasma cysteine is significantly lower in children with autistic disorder are dominated by one research group that published three studies (one in children with abnormal methylation or GSH:GSSG) [46-48] and their findings have been replicated by another research group [60]. The same study found no significant difference in plasma cysteine levels for children with PDD-NOS or Asperger's Syndrome, as did a study comparing autism spectrum disorders (sample composition unknown) compared to controls [77]. Plasma cysteine was significantly lower in a study comprising 28 children with autistic disorder and 10 children with PDD-NOS [91]. Serum cysteine levels of children with autistic disorder compared to controls were not significantly different from controls [69]. Overall statistical heterogeneity for plasma cysteine was considerable (I^2 ^= 92%) and low to moderate between diagnostic subgroups (I^2 ^= 39.8%). Again, factors that may have led to the high level of heterogeneity between studies include fasting status, differing laboratory methods and varied selection of controls.

**Figure 7:**
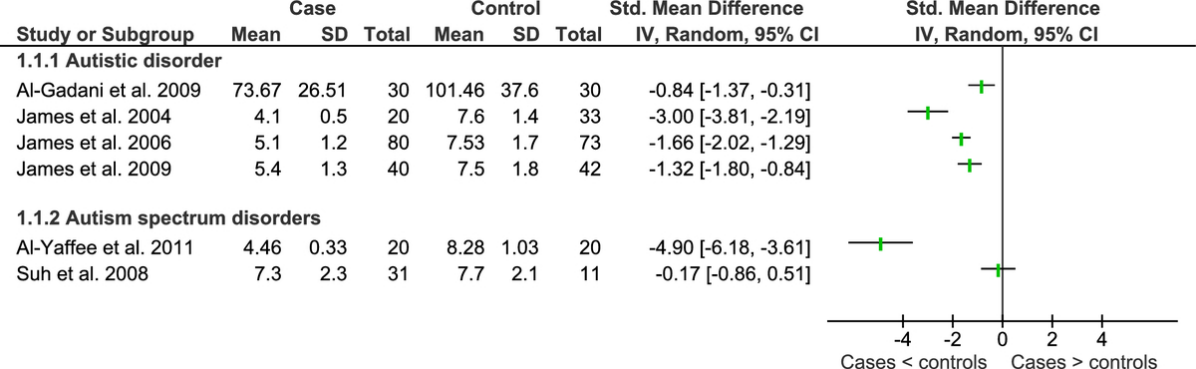
**Heterogeneity of studies that compared tGSH in children with autistic spectrum disorder and healthy controls**.

A significant decrease in plasma total glutathione (tGSH) reported in four studies from the one research group in children with autistic disorder compared to controls [46-48,50] have been confirmed by another two research groups with respect to autistic disorder [81,82] as well as study of low functioning children with autism spectrum disorders [85] (Figure 7). Reduced glutathione has also been reported to be lower in the plasma of children with autism spectrum disorders [87,91]. In contrast, no significant difference for plasma tGSH [77] or erythrocyte tGSH [65] was reported for autism spectrum disorders (mixed diagnoses). The later study compared cases to an adult reference range while noting that the paediatric range is lower. Whole blood tGSH was reported to be lower in autistic disorder but not significantly different for PDD-NOS or Asperger's Disorder [60]. Overall statistical heterogeneity was substantial for plasma tGSH (I^2 ^= 93%) however there was no statistical heterogeneity between diagnostic sub-groups (I^2 ^= 0%). Varying definition of cases and controls, laboratory and analytical methods may account for the range of heterogeneity.

**Figure 8:**
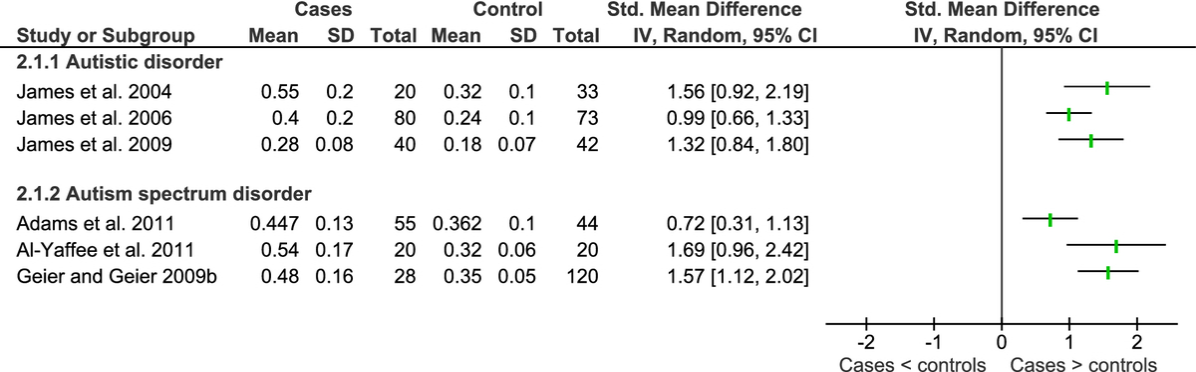
**Meta-analysis of studies that compared GSSG in children with autistic spectrum disorder and healthy controls**.

The same major research group published four studies showing a significant increase in plasma oxidised glutathione in autistic disorder [46-48,50] which has been replicated by a further two research groups for autism spectrum disorders [87,91] (Figure 8). Overall statistical heterogeneity was substantial (I^2 ^= 67%), however, there was no statistical heterogeneity between diagnostic subgroups (I^2 ^= 0%). Meta-analysis resulted in a SMD of 1.25 (95% CI 0.87 - 1.62) across 203 cases and 184 controls using a random effects model. As stated above, data from James *et al. *2009 was not included in the analysis but is included in the tables accompanying the Figure.

The same research group published 5 studies reporting significantly lower plasma tGSH:GSSG ratios in children with autistic disorder [46-50]. The same controls were used for three of the studies [47,49,50]. One of these studies tGSH and tGSH:GSSG were significantly lower (by 10.7% and 14.9% respectively) in children with autism who were heterozygous or homozygous for the delta aminolevulinic acid dehydratase (ALAD) 177 GC mutation, whereas there was no difference in GSSG [50]. This polymorphism is found in the heme biosynthesis pathway where it has been associated with altered toxicokinetics of lead levels and elevated blood levels of lead [100-102]. Three studies have shown that GSH:GSSG is lower in children with autism spectrum disorders [85,87,91] lending credence to the original findings.

The findings for cysteinyl-glycine, a breakdown product of glutathione, were inconsistent. Initially it was reported that there was no significant difference in cysteinyl-glycine in children with autistic disorder compared to controls [47]. A later study by the same research group was limited to children with abnormal methylation or GSH:GSSG showed that it had a significantly lower level in autistic disorder than in controls [48], however, a subsequent study of children with autism spectrum disorders showed that it was significantly higher than in controls [77]. Differences in inclusion criteria, laboratory methods and control of confounding variables may account for the difference in the findings.

Finally, the relationship between GSH and the immune system was clearly demonstrated in a large study which showed that children with autistic disorder universally have lower natural killer cell activity in peripheral mononuclear cells than those without the disorder which correlated with low intracellular levels of GSH as shown in Table 10[79]. Furthermore, when GSH was added to the culture medium, natural killer cell activity increased more in lymphocytes obtained from children with low natural killer cell activity than those with normal activity. Corresponding data were not provided for control children.

**Table 10:** Correlation between NK cell activity and reduced glutathione in peripheral blood mononuclear cells obtained from children with autistic disorder

Study	NK Activity (LU)	**GSH (ng/3 × 10**^**6 **^**PBMCs)**	Significance	Finding
Vojdani *et al. *2008	0-10	610 ± 286	ANOVA F = 3.99, *P *< 0.05	Direct correlation between cellular levels of reduced glutathione and NK lytic activity.
[79]	11-20	947 ± 458		
	21-50	1760 ± 895		
	51-100	2280 ± 1341		

NK Natural killer cells LU Lytic units GSH reduced glutathione ng nanograms PBMCs Peripheral blood mononuclear cells ANOVA One way analysis of variance

Glycine and glutamine are key compounds for the biosynthesis of glutathione obtained through dietary sources. Eight studies showed no significant difference in serum or plasma glycine in children with autistic disorder [60,69,70,75], autism spectrum disorders (mixed diagnoses) [86-88] PDD-NOS or Asperger's Syndrome compared to controls [60]. There was no statistical heterogeneity overall (I^2 ^= 0) or between diagnoses (I^2 ^= 0) for plasma glycine. The data was not pooled because there were only two studies for plasma and two for serum with data in a suitable form to combine.

In contrast the findings for glutamine were inconsistent with two studies reporting no significant difference in serum glutamine in children with autistic disorder compared with controls [69,89], a later study reporting a significant decrease in platelet glutamine in children with autistic disorder [68] and further studies that reported serum glutamate to be significantly higher in adults with autistic disorder [75] and children with autism spectrum disorders (mixed diagnoses) than controls [87].

Of the six studies that measured co-factors of the γ-glutamyl cycle or trans-sulphuration pathway, five studies showed elevated levels of vitamin B6 in children with autistic disorder or autism spectrum disorders compared to controls [73,76,87,92,93], one showed a decrease in erythrocyte selenium in children with autistic disorder [78] and another showed no change in whole blood selenium in children with an autism spectrum disorder compared to controls [87].

### Intervention studies

The findings of the six studies that report the outcome of interventions in autism focussed on normalising abnormalities in γ-glutamyl cycle or trans-sulphuration pathway metabolites [46,48,49,54,64] are presented in Table 11. An initial pilot study showed that supplementation of children with autistic disorder with 800 μg folinic acid and 1,000 μg betaine per day for two months normalised homocysteine levels and improved GSH:GSSG [46]. The addition of 75 μg/kg methyl-cobalamin injected twice weekly for one month further normalised GSH:GSSG. The same researchers conducted a larger intervention in 42 children with autistic disorder who had evidence of reduced methylation capacity or GSH:GSSG in which they were supplemented with folinic acid and methyl-cobalamin for 3 months [48]. The new regimen, which used half the dose of folinic acid of that used in the pilot study, resulted in significant increases in metabolites of the trans-sulphuration pathway as well as GSH:GSSG, although they remained below those of the control children. Objective behavioural measures showed an improvement, although all participants were still well below normal (data not published).

**Table 11:** *In vivo *studies involving interventions directed at normalising γ-glutamyl cycle or trans-sulfation pathway metabolites

Study	Intervention	Dose & duration	Study size	Findings	Comments
***Interventions involving folate cycle metabolites***					

Bertoglio *et al. *2010 [64]	Methyl-cobalamin	64.5 μg/kg methyl-cobalamin or placebo injected every third day 6 wks washout period).	32+ cases started the trial of which 30 finished.	Overall, no significant difference in GSH, GSH: GSSG or behaviouraloutcomes. ↑ GSH, ↑GSH:GSSG and improved behavioural outcomes in 9/30 children.	Primary outcome behavioural response. Findings for GSH and tGSH:GSSG presented as bar charts for responder group only. Dispersion and units not provided.

James *et al. *2009 [48]	Folinic acid + methyl-cobalamin	400 μg folinic acid bd + 75 μg/kg methyl-cobalamin injected every third day 3 mo.	48 cases selected for low SAM:SAH or GSH: GSSG.	↑ homocysteine, ↑ cystathione ↑ cysteine, ↑ tGSH & ↑tGSH: GSSG.	Excluded 26% of cases because normal SAM: SAH and/or tGSH: GSSG. Following the intervention, tGSH:GSSG was still significantly lower in cases than controls. There was no change in SAM or SAH levels.

James *et al. *2004 [46]	Folinic acid + betaine	800 μg folinic acid + 1,000 μg betaine bd 3 mo.	8 cases	↑ homocysteine, ↑ cystathione ↑ cysteine, ↑ tGSH, ↑tGSH: GSSG, ↓SAH & ↓adenosine	Improved but did not normalise tGSH and GSSH.
	Folinic acid + betaine +	As above + 75 μg/kg methyl-	8 cases	↑ homocysteine,	Normalised tGSH & tGSH:
	methyl-cobalamin	cobalamin injected twice weekly 1 mo.		↑cystathionine, ↑cysteine, ↑ tGSH, ↑tGSH: GSSG, ↓GSSG	GSSG. Improved but did not normalise GSSG.

Moretti *et al. *2005 [54]	Folinic acid	0.5 mg/kg/d folinic acid for 2 wk, 1.0 mg/kg/d thereafter 3 mo.	1 case	Normalised (↓) cerebral spinal fluid homocysteine.	

***Other interventions***					

Adams *et al. *2009 [65,67]	Chelation therapy	Glutathione (180 mg) or placebo cream daily for 7 days followed by 10 mg/kg DMSA in 3 doses/day for 3 days to screen for high urinary	64 cases	Significantly ↓ variance in erythrocyte glutathione levels 1-2 months after one round of DMSA treatment.	Topical glutathione had no effect on erythrocyte glutathione. Behavioural instruments not validated
		excretion of metal ions. 'High	26 DMSA	No data provided for	for measurement of autism
		excreters' from the topical	15 placebo	post intervention gluta-	severity. ADOS(diagnostic
		glutathione arm given a further		thione.	test) administered pre and
		6 rounds of DMSA and those			post second intervention,
		from the topical placebo arm			but not at baseline.
		given 6 rounds of a placebo.			

Rossignol *et al. *2007 [49]	Hyperbaric oxygen therapy (HBOT)	1.3 atm (n = 12) or 1.5 atm (n = 6) for 45 mins × 40 sessions (ie 4.6 times/wk × 9 wk)	18 cases	No significant difference in plasma tGSH:GSSG before or after either intervention.	

SAM s-adenosyl-methionine SAH s-adenosyl-homocysteine tGSH total glutathione GSSH Oxidised glutathione bd twice daily DMSA dimercaptosuccinic acid ADOS autism diagnostic observation schedule

Recently, a double blinded randomised controlled trial was published in which participants were administered either methyl-cobalamin or placebo for 6 weeks and then their treatment switched without washout for a further 6 weeks [64]. Overall, there was no significant change in GSH, GSH:GSSG or behaviour. Thirty percent of participants, however, showed a significant improvement in objective behavioural measures which correlated with improved plasma GSH and GSH:GSSG levels. Interpretation of the findings is difficult because data was only provided for the 'responder' subgroup and this did not include standard deviations or units for plasma GSH or GSH:GSSG, nor did it state whether the GSH values reported in Figure 4 of their paper represented tGSH or reduced glutathione. Furthermore, data showing whether 'responders' had lower baseline concentrations of GSH or GSH:GSSG were not provided.

Additionally, a case report of a child with autistic disorder and cerebral folate deficiency showed the normalisation of low cerebral spinal fluid homocysteine following 2 weeks supplementation with 0.5 mg folinic acid/kg/day increasing to 1.0/kg/day for 3 months [54]. Finally, a 40 session trial of hyperbaric oxygen therapy showed that it has no effect on plasma GSH:GSSG in children with autistic disorder [49] and, as discussed above, incomplete data and selective reporting make it hard to interpret the findings of a randomised trial of topical glutathione before chelation [65].

### Genetic studies of the γ-glutamyl cycle and trans-sulphuration pathway

The six studies that presented data on genetic polymorphisms of the γ-glutamyl cycle or trans-sulphuration pathway are summarised in Table 12. The best powered of these studies examined genetic variation in 42 genes (308 single nucleotide polymorphisms (SNPs)) related to glutathione, including those coding for enzymes that use glutathione as a co-factor (not included in this review), in 318 families from the Autism Genetic Resource Exchange repository [87]. Several SNPs located in the genes for cystathionine γ-ligase (CTH), alcohol dehydrogenase 5, GCL and glutaredoxin showed significant or suggestive associations with autism spectrum disorders. Interaction models confirmed a significant association between CTH, glutaredoxin and glutaredoxin 3 and autism (OR = 3.78 (95% CI 2.36-6.04).

**Table 12:** Genes associated with the α-glutamyl cycle or trans-sulfation pathway

Study	Study Size	Population	Gene	Polymorphism	P value	Finding
***Glutathione-s- transferases***						

***GST-M1***						

Buyske *et al. *2006 [96]	172 controls (54 case parent trios)	U.S. (non-Hispanic Caucasians)	GST-M1	GST-M1*0	0.028 (Pearson's) 0.046(Likelihood ratio)	Homozygotes with deletion at increased risk.

James *et al. *2006 [47]	360 cases 205 controls	U.S. (97% Caucasian)	GST-M1	GST-M1*0	0.067^1,2^	Suggestive increase of null genotypes in cases.

Bowers *et al. *2011 [94]	318 families (1,149 individuals)	U.S.	GST-M1	tag SNPs	ns	No association.

James *et al. *2006 [47]	As above	As above	GST-M1 +	GST-M1*0: RFC	0.00014^1,2^	This combination more frequent

			RFC	80A > G interaction		in cases. OR 3.78(95%CI 1.80, 7.95).

***GST-P1***						

Serajee *et al. *2004 [97]	196 case parent trios	U.S.	GST-P1	rs947894	ns	

Bowers *et al. *2011 [94]	As above		GST-P1	tag SNPs	ns	

***GST-T1***						

James *et al. *2006 [47]	As above	As above	GST-T1	GST-T1*0	ns^2^	

***Glutathione peroxidase***						

Ming *et al. *2010 [95]	101 cases (results	U.S	GPx-1	GCG repeat		
	based on 68 trios			ALA5	ns	
	and 3 duos)			ALA6	0.017	Under-transmitted
				ALA7	ns	

Bowers *et al. *2011 [94]	As above	As above	GPx-1	tag SNPs	ns	

***ALAD***			***Single SNP analysis***			

Rose *et al. *2008 [50]	451 cases	U.S.	ALAD	rs1800435	0.023	GC OR 1.65 (1.05-2.63)

	251 controls				0.6	CC OR 1.82 (0.14-95.73)

			***Interaction analysis***			

			ALAD rs1800435*GC			ALAD/RFC GG/AA reference

			RFC 80A > G combined		0.162	ALAD/RFC GC/AA OR 2.25 (0.72-7.06)

			genotype associated with		0.001	ALAD/RFC GCAG OR 3.98 (1.70-9.32)

			increased risk of autism.		0.237	ALAD/RFC GC/GG OR 1.79 (0.68-4.73)

**Study**	**Study Size**	**Population**	**Gene**	**Polymorphism**	**P value**	**Finding**

***Other relevant genes***			***Single SNP analysis***			

Bowers *et al. *2011 [94]	As above		CTH	rs12737233	0.002 *(0.30)*^3^	CT OR 0.91 (0.65-1.28)
	*(Validation study in 3327 individuals from independent*					TT OR 4.83 (1.85-12.59)
	*AGRE families)*		GCLC	rs761141	0.02 *(0.10)*^3^	CT OR 2.10 (1.20-3.69) TT OR 1.67 (0.91-3.09)
				rs524553	0.003 *(0.08)*^3^	CT OR 2.70 (1.47-4.98)
						TT OR 2.23 (1.16-4.28)
			DPP-1	tag SNPs	ns	
			DPP-2	tag SNPs	ns	
			DPP-3	tag SNPs	ns	
			GGT-7	tag SNPs	ns	
			GGT-5	tag SNPs	ns	
			GGT-LA4	tag SNPs	ns	
			GPx-2	tag SNPs	ns	
			GPx-4	tag SNPs	ns	
			GPx-7	tag SNPs	ns	
			GST-A2	tag SNPs	ns	
			GST-A3	tag SNPs	ns	
			GST-A4	tag SNPs	ns	
			GST-K1	tag SNPs	ns	
			GST-M2	tag SNPs	ns	
			GST-M3	tag SNPs	ns	
			GST-M4	tag SNPs	ns	
			GST-M5	tag SNPs	ns	
			GST-O1	tag SNPs	ns	
			GST-T2	tag SNPs	ns	
			GST-Z1	tag SNPs	ns	
			GST-CD	tag SNPs	ns	
			***Interaction analysis***			
			CTH rs12737233*C			OR 3.78 (95%CI 2.36-6.04)
			GLRX3 rs370834*A			
			GLRX rs17216887*G			
			allele combination associated			
			with increased risk of autism.			

ns not significant GSTP1 Glutathione-S-transferase P1 GSTM1 Glutathione-S-transferase M1 RFC Reduced folate carrier SHMT Serine hydroxyl methyl transferase GPx-1 Glutathione peroxidase-1 ALA Poly-alanine repeat polymorphism CTH Cystathione gamma lyase GCLC γ-cysteine synthase, catalytic sub-unit GST-CD Glutathione-s-transferase, C terminal domain GGT-7 γ-glutamate transferase 7 GGT-5 γ-glutamate transferase 5 GGT-LA4 γ-glutamate transferase like activity 4 DPP Dipeptidase GLRX3 Glutaredoxin 3 GLRX Glutaredoxin SNP Single nucleotide polymorphism

^1 ^Calculated by authors using EpiInfo version 6.0 Statcalc ^2 ^Uncorrected ^3^P value from validation study in independent AGRE families

The study found no association between any of the GST genes and autism [94]. This is in contrast to a previous study of case parent trios that found that homozygote cases for the GST-M*1 gene deletion (GST-M1*0) showed increased risk of autistic disorder [96] and a later case control study that reported a borderline association between the GST-M*1 gene deletion (GST-M1*0) and autistic disorder and a significant interaction between the GST-M1*0 deletion and the reduced folate carrier 80A > G [47]. Previous studies failed to find an association between GST-T1 [47] or GST-P1 and autism [97].

Ming et al. 2010 Found that a polyalanine repeat polymorphism in the GPx gene (*GPx-1*) was associated with autistic disorder [95]. Under-transmission of the variation encoding six alanine residues (ALA6) was observed in the families with autistic disorder, suggesting that this allele may be protective. The authors acknowledge that their interpretation is limited by inadequate knowledge of the function of the ALA alleles of *GPx-1 *gene.

### Studies of glutathione related enzyme activity

As shown in Table 13 GPx-1 activity has been the subject of seven studies [63,71,72,74,81,83,84]. The findings were inconsistent in plasma where two studies reported higher activity in cases than controls [72,81] and two reported lower activity [71,83]. A further four studies examined GPx-1 activity in erythrocytes. Of these, two reported lower activity [63,71] and two reported no significant difference between cases and controls [74,84]. No significant difference between cases and controls was reported for GPx-1 activity in platelets [63]. In addition, a recently published study showed that glutathione-S-transferase activity was significantly reduced in children with low functioning autism spectrum disorders and there was a trend towards lower activity of glutathione reductase [85].

**Table 13:** Studies examining enzymes of the γ-glutamyl cycle or transulphuration pathway in autism

Source	Study	Study size (Male:Female)	**Cases**^**1**^	**Controls**^**1**^	*P *values	Overall finding	Units of measure
***Glutathione peroxidase***							

Plasma	Yorbik *et a*l. 2002 [71]	45 cases, (39 M, 6 F) 41 controls, (35 M, 6 F)	270 ± 40	390 ± 80	< 0.05	low	U/L

	Söğüt *et al. *2003 [72]	27 cases, (16 M, 11 F) 30 controls, (16 M, 14 F)	40.9 ± 11.3	24.2 ± 6.3	< 0.0001	high	U/L

	Al-Gadani *et al. *2009 [81]	30 cases, (22 M, 8 F) 30 controls, (20 M, 10 F)	2468.8 ± 999.3	1438.5 ± 611.2	< 0.05	high	U/L

	Mostafa *et al. *2010 [83]	44 cases, (30 M,14 F) 44 controls, (30 M, 14 F)	441.5 (100)^2^	589 (176)^2^	< 0.001	low	U/L

Erythrocytes	Golse *et al. *1978 [63]	24 cases, (17 M, 7 F) 6 controls, (2 M, 4 F)	4.7 ± 0.29	8.45 ± 0.95	< 0.0001	low	U/g haemoglobin

	Yorbik *et al. *2002 [71]	45 cases, (39 M, 6 F) 41 controls, (35 M, 6 F)	28.72 ± 2.64	38.01 ± 5.03	< 0.05	low	U/g haemoglobin

	Pasca *et al. *2006 [74]	12 cases, (9 M, 3 F) 9 controls, (6 M, 3 F)	7.75 ± 0.93	7.45 ± 0.65	ns	ns	U/g haemoglobin

	Vergani *et al. *2011 [84]	28 cases (21 M, 7 F) 32 controls (20 M, 12 F)			ns	ns	nmols NADPH/min/mg haemoglobin

Platelets	Golse *et al. *1978 [63]	36 cases, (15 M, 21 F) 21 controls, (9 M, 12 F)	51.53 ± 1.61	46.56 ± 2.69	ns	ns	U/g platelet protein

***Glutathione reductase***							

Plasma	Al-Yafee *et al. *2011 [85]	20 cases (20 M, 0 F) 20 controls (20 M, 0 F)	70.25 ± 16.35	60.19 ± 15.42	0.052	trend high	U/L

***Glutathione-s-transferase***							

Plasma	Al-Yafee *et al. *2011 [85]	20 cases (20 M, 0 F) 20 controls (20 M, 0 F)	0.42 ± 0.18	0.73 ± 0.37	0.002	low	μmol/min/ml

ns Not significant ^1^Mean ± standard deviation ^2 ^Median and interquartile range

## Discussion

The findings of this systematic review support the assertion that children with autism spectrum disorders are more likely to have significantly lower tGSH and GSH and significantly increased GSSG, resulting in a significantly lower GSH:GSSG than children without autism. Our review show that although serum homocysteine and cystathione levels are not significantly different in children with autism spectrum disorders compared to those without, serum cysteine is significantly lower in autistic disorder (Figures 4, 5 and 6) which supports the assertion that cysteine production may be the rate limiting step in glutathione synthesis [31-34]. The lack of an association in other forms of autism spectrum disorders suggests that low cysteine may be associated with autism severity [60,77].

As no significant differences in serum homocysteine, cystathionine (Figures 4 and 5) or serine levels [60,69,70,75] were observed in children with autistic disorder compared to those without, it can be inferred that the low levels of cysteine may be caused by decreased cystathione lyase activity and/or increased utilisation of sulphate and/or taurine and/or lower dietary intake or absorption of cysteine in children with autistic disorder. An exploration of the functional significance of several SNPs in the gene for cystathione lyase associated with autistic disorder may shed light on this putative relationship [94].

It is worth noting that a reduction in total glutathione implies a problem with synthesis of glutathione, whereas a decrease in GSH implies an anomaly in GSH:GSSG. Both have been observed in autism spectrum disorders [46-48,50,77,81,82,85,87,91]. As the bioavailability of cysteine is the rate limiting factor for synthesis of GSH [31-34], the lower cysteine levels detected in serum of children with autistic disorder could be an important factor leading to the lower levels of GSH observed in many children with this condition [46-48,50,81,82]. While several studies examined GPx activity [63,71,72,74,81,83,84] or its polymorphisms [94,95], only one examined glutathione reductase or glutathione-S-transferase activity [85].

The higher level of GSSG observed in the serum of many children with autism spectrum disorders [46-48,50,85,87,91] is likely to truly reflect increased oxidative stress as there is no significant difference in GPx-1 activity in serum or platelets [63,84] and GPx-1 is significantly lower in the erythrocytes of children with autistic disorder compared to controls. As cysteine itself may have strong anti-oxidant properties, its lower concentration in children with autistic disorder may contribute to increased oxidation of GSH [47].

Glutathione synthesis also requires dietary glutamate and glycine. No difference in serum levels of glutamate has been reported between children with autistic disorder and controls [69,70], however, it was significantly higher in adults with autistic disorder [75], the level being positively correlated with social functioning as measured by the autism diagnostic interview (ADI-R) [103] which is commonly used to evaluate the core symptoms of autism which may shed light on the significantly higher level detected in mixed samples of children with autism spectrum disorders [86,87]. In contrast, platelet glutamic acid was significantly lower in children with autistic disorder [68]. Platelet glutamate receptors have been shown to have heightened sensitivity in major depression, schizophrenia and other psychoses [104-106]. This relationship has not, however, been examined in autism spectrum disorders to date.

Serum and plasma glycine does not differ between children [60,69,70,86,87] or adults [107] with and without autism spectrum disorders. As glycine acts as a powerful inhibitory neurotransmitter in the brain and spinal chord [108] and glycine transporters are differentially expressed throughout the CNS [109], it may be relevant for future studies of children with autism spectrum disorders to examine whether there are abnormalities in expression of glycine transporters in the CNS or in glycine transport across the choroid plexus in children with autism spectrum disorders.

Vitamin B6, in the form of pyridoxal-5'-phosphate, is the cofactor for 5 enzymes in the γ-glutamyl cycle and trans-sulphuration pathway: cystathionine β-synthase, cystathione γ-lyase, cytoplasmic and mitochondrial serine hydroxymethyltransferase and glycine decarboxylase in the mitochondria. The significant increase in plasma vitamin B6 in many children with autism spectrum disorders [73,76,87,92,93] could potentially reflect diminished cellular uptake or inefficiency of cells to retain or store B6. Impaired bioavailability of vitamin B6 may affect the nervous system because it is required for the synthesis of neurotransmitters including serotonin, dopamine and taurine [110]. There is clearly a need to test whether cellular vitamin B6 is diminished in autism spectrum disorders. In addition, erythrocyte selenium was shown to be significantly lower in one study of children with autistic disorder [78] although there was no significant difference in GPx activity which contains selenium in its active site. These observations coupled with the findings of the intervention studies [46,47] suggest that future studies should examine multiple nutritional status biomarkers of pathways linked with the γ-glutamyl cycle (e.g. cysteine, folate, vitamin B12, vitamin B6) to determine more effective and accurate risk factor analysis.

The association between the genes of the γ-glutamyl cycle and autistic disorder is not well studied, although recently a relatively large study using a pathway approach showed a three-SNP joint interaction effect for glutaredoxin, glutaredoxin 3 and cystathione lyase (OR = 3.78, 95% CI: 2.36, 6.04) as well as marginal associations for cystathione lyase, the gamma-glutamylcysteine synthetase, catalytic subunit and glutaredoxin 3 suggesting that variation in genes involved in counterbalancing oxidative stress may contribute to autism [94]. The clinical significance of the borderline association between the GST-M*1 gene deletion (GST-M1*0) and autistic disorder [47,96] and the interaction between the GST-M1*0 deletion and the reduced folate carrier 80A > G [47] has yet to be established. GST-M1*0Frequencies range from 42-60% in Caucasians [111] and GST-M1*0 appears to be associated with social behavioural changes in mice compared to wild type [112]. No significant associations were found for GST-T*1 [47]. Although no association between *GST-P1 *has been shown in children with autism [94,97], the GSTP1*A haplotype was over-transmitted

to mothers of children with autistic disorder (OR 2.67 95% confidence interval, 1.39- 5.13) [113]. Bowers *et al *2011 did not find an association with autistic disorder and other members of the two GST super-families, many of which have GPx activity [22].

The observation that cytostolic and mitochondrial glutathione redox ratios are significantly lower in lymphoblastoid cell lines obtained from children with autistic disorder than their non-autistic siblings [80] coupled with a positive correlation between low natural killer cell activity and reduced glutathione levels in children with autistic disorder [79] suggests that these cells may be more GSH dependent. It is reasonable to hypothesise that children with autistic disorder could benefit from supplementation with glutathione or its precursors. Studies are needed to establish the relationship between extracellular and intracellular glutathione in its oxidised and reduced form because intracellular may ultimately be a better biomarker to study cellular effects proposed in theoretical models.

Finally, phenotypic approaches have been taken to study resistance to environmental or endogenous stressors that are thought to be causative agents for other diseases (e.g. cancer) using lymphocytes or fibroblasts [114-118]. This approach has the advantage that it integrates the effect of both genetic background and nutritional status of the cells in evaluating disease susceptibility. The only published study applying this approach to date in autistic disorder found that the GSH:GSSG was significantly lower in whole cell extracts and mitochondria from lymphoblastoid cells obtained from children with autistic disorder compared to controls but there was no difference in response to nitrosative stress as measured by GSH:GSSG in mitochondria [80].

The consistent observations indicating a role for glutathione metabolism in autism spectrum disorders highlight the potential role of glutathione in them. Replication of these results by other research groups in a wider range of populations is required, however, before definite conclusions can be made. Meta-analyses of observational studies are subject to confounding and selection bias which can distort the findings to produce spurious results [119]. We have been careful not to make our meta-analyses a prominent component of the review, but rather to use the data to identify the extent of confounding and selection biases and to examine possible sources of heterogeneity between the results of individual observational studies.

Common challenges for any systematic review of studies investigating autism spectrum disorders include changes in the criteria for diagnosis and laboratory methodology, the emphasis of early studies on individual metabolites rather than metabolic systems (e.g. one carbon metabolism), and low statistical power. Only a few studies matched cases and controls for age [60,68,74,75,83] and/or gender [48,60,75,83,89] despite the acknowledged difference in prevalence between genders [99], and most did not state the source of controls [48,59,60,63,68-70,73,74,76,78,81,84].

Only two of the reviewed studies corrected for multiple comparisons (e.g. Bonferroni or Nyholt correction) [86,97]. The few studies that matched cases with controls did not use a paired method of analysis such as McNemar's test which biases the results towards null. Further, we were unable to replicate the p values for three studies [46,47,87].

## Conclusions

This review of published studies indicates that evidence for the involvement of the γ-glutamyl cycle and trans-sulphuration pathway in autism spectrum disorders should be further explored with higher quality and lower risk bias studies to in order to ascertain the significance of these pathways with respect to clinical outcomes. Trends noted from currently published studies include decreased total glutathione and increased GSSG in children with autism spectrum disorders with no association found between homocysteine or cystathione in these children. There is a trend for cysteine to be lower in children with autistic disorder but not in other autism spectrum disorders. There is a need for large, well designed studies that link metabolites, co-factors and genes of the γ-glutamyl cycle and trans-sulphuration pathway with objective behavioural outcomes to be conducted in children with autism spectrum disorders. Future risk factor analysis of autism spectrum disorders should include consideration of multiple biomarkers of nutrients involved in pathways linked with the γ-glutamyl cycle and the interaction of genotype with nutritional status.

## Abbreviations

ADI-R: Autism diagnostic interview - revised; ALAD: Delta aminolevulinic acid dehydratase; CARS: Childhood Autism Rating Scale; CNS: Central nervous system; d.f.: Degrees freedom; DSM: American Psychiatric Association's Diagnostic and Statistical Manual of Mental Disorders; fGSH: Free reduced glutathione; GCL: Glutamate cysteine ligase; GCT: γ-glutamyl cyclotransferase; GGT: γ-glutamyl-transferase; GPx: Glutathione peroxidase; GSH: Reduced glutathione; GSH:GSSG: Glutathione redox ratio; GSH-R: Glutathione reductase; GS: Glutathione synthetase; GSSG: Oxidised glutathione; GST: Glutathione-S-transferases; Mg^2+^: Magnesium ions; Mn^2+^: Manganese ions; Q: Cochran's Q (a measure of statistical heterogeneity); SMD: Standardised mean difference; tGSH: Total glutathione

## Competing interests

The authors declare that they have no competing interests.

## Authors' contributions

PM was responsible for all aspects of the manuscript. MA, CO'D and PT checked the references, PM and CO'D assessed the quality of included studies and the draft was reviewed by MA, CO'D, PT and MF. All authors read and approved the final manuscript.
